# Cell‐Based Therapies for Alveolar Bone and Periodontal Regeneration: Concise Review

**DOI:** 10.1002/sctm.19-0183

**Published:** 2019-11-06

**Authors:** Federico Moreno Sancho, Yago Leira, Marco Orlandi, Jacopo Buti, William V. Giannobile, Francesco D'Aiuto

**Affiliations:** ^1^ Unit of Periodontology UCL Eastman Dental Institute London United Kingdom; ^2^ Medical‐Surgical Dentistry (OMEQUI) Research Group Health Research Institute of Santiago de Compostela Santiago de Compostela Spain; ^3^ Department of Periodontics and Oral Medicine University of Michigan School of Dentistry Ann Arbor Michigan USA; ^4^ Department of Biomedical Engineering, College of Engineering and Biointerfaces Institute University of Michigan Ann Arbor Michigan USA

**Keywords:** Bone, Alveolar ridge preservation, Lateral alveolar ridge augmentation, Sinus augmentation, Periodontal regeneration, Cell‐ and tissue‐based therapy

## Abstract

Current regenerative strategies for alveolar bone and periodontal tissues are effective and well adopted. These are mainly based on the use of a combination of synthetic/natural scaffolds and bioactive agents, obviating the incorporation of cells. However, there are some inherent limitations associated with traditional techniques, and we hypothesized that the use of cell‐based therapies as part of comprehensive regenerative protocols may help overcome these hurdles to enhance clinical outcomes. We conducted a systematic review of human controlled clinical trials investigating the clinical and/or histological effect of the use of cell‐based therapies for alveolar bone and periodontal regeneration and explored the translational potential of the different cell‐based strategies identified in the included trials. A total of 16 studies (11 randomized controlled trials, 5 controlled clinical trials) were included for data synthesis and qualitative analysis with meta‐analyses performed when appropriate. The results suggest a clinical benefit from the use of cell therapy. Improved outcomes were shown for alveolar ridge preservation, lateral ridge augmentation, and periodontal regeneration. However, there was insufficient evidence to identify best‐performing treatment modalities amongst the different cell‐based techniques. In light of the clinical and histological outcomes, we identify extraction socket and challenging lateral and vertical bone defects requiring bone block grafts as strong candidates for the adjuvant application of mesenchymal stem cells. Given the complexity, invasiveness, and costs associated with techniques that include “substantial manipulation” of tissues and cells, their additional clinical benefit when compared with “minimal manipulation” must be elucidated in future trials. stem cells translational medicine *2019;8:1286&1295*

## Graphical abstract

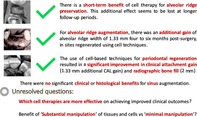

**Significance Statement**

Cell‐based therapies have the potential to improve outcomes of regenerative treatment in the oral cavity. Extraction sockets and challenging lateral/vertical bone defects requiring bone block grafts are ideal candidates for the adjuvant application of mesenchymal stem cells. The present review showed that there is insufficient evidence to identify best‐performing treatment modalities among the different cell‐based techniques. Given the complexity, invasiveness, and costs associated with the “substantial manipulation” of tissues and cells, the clinical benefit of these techniques when compared with “minimal manipulation” must be elucidated. Further research evaluating the effectiveness of simple, fast, and economical methods for cell harvesting and processing is warranted.

## Introduction

Outcomes in alveolar bone and periodontal regeneration are largely dependent on the biological and the material characteristics of scaffolds and the availability, recruitment and activation of cells and biomolecules in the injured area during healing. A large number of reviews have assessed the outcome of bone regeneration techniques either before or at the time of implant placement and following maxillary sinus augmentation 1, 2, 3, 4, 5, 6, 7, 8, 9, including Cochrane systematic reviews 10, 11. The efficacy of different surgical techniques and biomaterials for periodontal regeneration has also been extensively documented 12, 13. These therapies are relatively simple and highly cost‐effective and, as a result, the use of autologous bone and/or bone substitutes, resorbable and non‐resorbable membranes, and different commercially available bioactive products are now routine practice across the globe. Although proven effective, there is also evidence that there is large degree of variability on the outcomes achieved. Failures and high complication rates are common in general dental practice in larger horizontal and vertical alveolar bone defects because the augmentation procedures are highly technique‐sensitive. Furthermore, the use of slow resorbing bone biomaterials may reduce the amounts and quality of newly formed bone in the augmented area. These issues delay treatment delivery significantly, especially for the replacement of teeth with dental implants shortly after tooth extraction. The addition of “smart” bioactive agents and cell‐based approaches to bone scaffolds and membranes may help overcoming some of these limitations. The opportunity lies on helping surgeons' activity through advanced but simple and fast protocols of tissue engineering.

The primary aim of this systematic review is to assess the efficacy of cell therapies on clinical and histological outcomes for alveolar bone and periodontal regeneration. We will focus on the use of these innovative approaches in human clinical studies investigating specifically ridge preservation, horizontal and vertical alveolar bone augmentation, sinus augmentation techniques, and periodontal regenerative surgeries. The results will be stratified according to the defect type but also with regard to the source of mesenchymal stem cells (MSCs) and the techniques for manipulation of tissue samples and cells. As a secondary outcome, we aim to investigate the translational potential outside a hospital/university setting of the different cell‐based strategies identified in the included controlled clinical trials.

## Methods

The protocol was registered on the PROSPERO database: ID CRD42019121119 (www.crd.york.ac.uk/PROSPERO).

### Objectives

- To investigate the evidence for the effect of cell‐based therapies as adjuncts to surgery for the regeneration of alveolar bone/periodontium.
- Critically appraise the validity, methodology, and outcomes of the included studies.
- Identify and appraise the clinical applicability of emerging cell‐based techniques for alveolar bone/periodontal regeneration.

### Types of studies

The review included prospective controlled clinical studies in human adults (18 years of age or above) evaluating the effect of surgical treatment with or without the adjunctive use of cell‐based therapies for the treatment of alveolar bone/periodontal defects assessing changes in alveolar bone dimensions/periodontal clinical measurements. Secondary outcomes were assessed only for those studies reporting data on the primary outcomes. A minimum of 6 weeks follow‐up post‐surgically for alveolar ridge preservation, 3 months follow‐up for lateral/vertical ridge, and sinus augmentation and 12 months for periodontal regeneration studies were required. Studies were stratified according to the defect model and each group was analyzed independently: (a) ridge preservation (ARP); (b) lateral and/or vertical ridge augmentation (GBR); (c) sinus augmentation (SINUS) d) periodontal regeneration (PERIO). Studies investigating the use of cell therapies to reconstruct large defects due to maxillectomies were excluded. Case reports and/or case series, retrospective clinical studies, and publications reporting the outcomes of in vitro studies or preclinical (animal) studies only were excluded.

### Determination of Outcome Measures

The primary outcome was dependent on the defect model: postsurgical alterations in the alveolar bone defect size, based on direct/indirect clinical and/or radiographic measurements were considered as primary outcomes. See the Supporting Information for all primary and secondary outcomes selected according to the defect type.

### Search Strategy

A highly sensitive search was conducted. Electronic databases (MEDLINE, EMBASE, LILACS) were searched using a string of medical subject heading and free‐text terms. OpenGrey was searched for the unpublished, gray literature. The search strategy was first designed for the MEDLINE database and then modified appropriately for the other databases (see Supporting Information). There were no language or publication date restrictions. Reference lists of all studies included for full text screening and previous reviews were searched for missing records. A manual search for the period from December 2013 to May 2019 was completed for a number of scientific journals (see Supporting Information). Further details regarding study selection, data extraction and analysis, quality assessment, and the processes followed for data synthesis can be found in the Supporting Information.

## Findings

### Search

A total of 5,053 potentially eligible records were returned from the electronic searches. A further 39 publications were found through the manual search of the selected journals. Following deletion of duplicates, a total of 4,741 records were available for initial title/abstract screening, after which the full‐text of 74 publications were retrieved. After assessment of the full papers, a further 56 records were excluded. Therefore, a total of 18 14, 15, 16, 17, 18, 19, 20, 21, 22, 23, 24, 25, 26, 27, 28, 29, 30, 31 publications corresponding to 16 original studies were included for qualitative analysis. One of the included studies reported follow‐up data from a previously published study 30, whereas another one was a correction of a previous report 31. The reason for exclusion of all studies which were not included following full text assessment was documented (see Supporting Information). Agreement between examiners after full‐text screening was excellent (Kappa score: 0.896).

**Figure 1:**
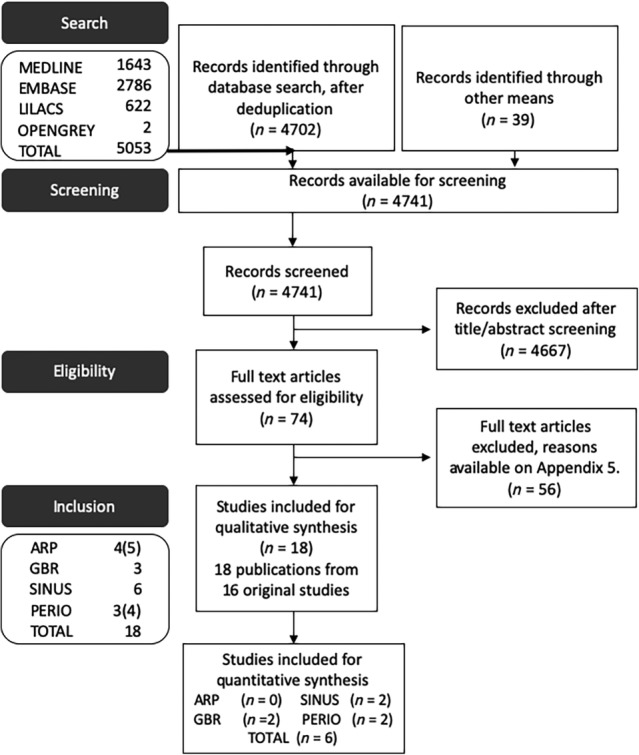
Flow diagram (following PRISMA guidelines) of screening and selection process.

There was a total of 11 randomized controlled trials (RCTs) 16, 17, 18, 19, 20, 24, 25, 26, 27, 28, 29 and 5 non‐randomized controlled original trials 14, 15, 21, 22, 23 included for qualitative analysis. All RCTs incorporated a parallel design, whereas CCTs included reports with either a parallel or a “split mouth” design. Chief characteristics of the included studies stratified according to defect type can be found in the Supporting Information. Outcomes were assessed through an array of different methods. In alveolar bone regeneration studies (ARP, GBR, SINUS), the method of measurement of the alveolar ridge dimensions (width and/or height) varied between studies and ranged from direct linear measurements measured in situ with a probe to linear and volumetric changes assessed radiographically (Orthopantomogram and Cone Beam Computed Tomography). Furthermore, analysis of core bone biopsies via histology, microCT, and Synchroton X‐ray Holotomography was also reported. The biopsy assessments mainly quantified new bone formation and relative ratios of new bone, graft remnants, and marrow space. Secondary outcomes frequently reported included complication rates, but only two studies reported other clinically relevant assessments such as the need for additional bone grafting at the time of implant placement and amount of additional bone graft needed 16, 17. For periodontal regeneration, outcome measures included Clinical Attachment gain (CAL), probing depth reduction (PD), Recession (REC), and defect bone fill (BF) (Fig. 1).

We identified cell‐based therapies according to the type of MSCs harvested and the donor tissue. Seven studies reported the use of bone marrow stem cells (BMSCs) harvested from posterior iliac crest aspiration 16, 17, 18, 19, 20, 21, 25, 26, two studies investigated the performance of dental pulp stem cells (DPSCs) harvested from the dental pulp of extracted teeth 14, 28, five of the included publications involved the harvesting of periosteum‐derived stem cells (PdSCs) from gingival connective tissue samples 15, 21, 22, 24, 29, one report produced periodontal ligament‐derived stem cells (PdlSCs) “cell sheets,” 27 and another publication used the adipose‐stromal vascular fraction from adipose tissue samples obtained via lipo‐aspiration 23. In line with the definition from the European Medicines Agency Regulation (EC) no 1394/2007 32, we further subcategorized the techniques in another two groups: (a) “minimal manipulation” of tissues/cells or (b) “substantial manipulation” otherwise (Table 1). More details about the cell‐scaffold combinations and the specifics of the different cell‐based harvesting techniques used in all the included publications are presented in the Supporting Information.

**Table 1:** Key features of cell harvesting and manipulation techniques described in the included controlled trials

Cells	Source	Harvesting invasiveness	Manipulation (proprietary system)	Defect/studies		Study type /RoB	External resources	Time	Cost	Clinical outcome	Histology outcomes
BMSCs	Bone marrow aspirate	Moderate Iliac crest punch LA /conscious sedation	Substantial (Replicell)	ARP / SINUS/	Kaigler et al. 2013 Kaigler et al. 2015	RCT/SC RCT/SC	Multiple hematologist and GMP lab	>12 days	Very high	Superior/large^a^ No difference	Enhanced^a^ Similar
			Minimal (BMAC) ^1^(harvest Terumo) ^2^(BMAC)	ARP / GBR /	Pelegrine et al. 2010 Correa et al. 2013^1^ Da Costa et al. 2011 Pelegrine et al. 2016^2^ Sauerbier et al. 2011^2^	RCT/high RCT/high RCT/high RCT/high RCT/high	Hematologist	Hour(s)	High	Superior/large^b^ No difference Superior/modest No difference Superior/modest	Similar Similar Enhanced^c^ Similar Poorer
DPSCs	Dental pulp, extracted teeth	High, not readily available	Substantial (Rigenera)	ARP/	D'Aquino et al. 2009	CCT/high	GMP lab	21 days	Very high	Superior/modest	Enhanced^d^
			Minimal (Rigenera)	PERIO/	Ferrarottiet al. 2018	RCT/low	None	Minutes	Very low	Superior/large	—
PdSCs	Periosteum, gingival connective tissue sample	Very low	Minimal (Rigenera)	ARP / SINUS/	D'Aquino 2016 Ceccarelli et al. 2018	CCT/high RCT/high	None	Minutes	Very low	Superior/modest —	— —
			Substantial (cell sheets)	SINUS/ PERIO/	Nagata et al. 2012 Ogawa et al. 2016 Yamamiya et al. 2008	CCT /high CCT/high RCT/SC	GMP lab	>6 weeks	Very high	No difference No difference Superior/large	Enhanced^d^ — —
PdlSCs	Periodontal ligament, extracted teeth	High, not readily available	Substantial (cell sheets)	PERIO/	Chen et al. 2016	RCT /low	GMP lab	4‐5 weeks	Very high	Superior/modest	—
A‐SVF	Adipose tissue, Lipo‐aspiration	High, liposuction/GA	Minimal (Celution)	SINUS/	Prins et al. 2016	CCT /SC	Plastic surgeon	Hour(s)	High	No difference	Similar

Replicell (Aastrom biosciences) is a commercial automated cell processing unit for isolation of a mixed population of CD90+ MSCs, hematopoietic SCs, inflammatory and endothelial cells (TRCs). Harvest Terumo BCT (Terumo medical do Brasil) is a commercially available system to obtain BMAC including a centrifuge (SmartPrep2) and a processing kit. BMAC system (Harvest Technologies) is a commercially available system to obtain BMAC which includes a harvest BMAC process disposable, 11‐ and 15‐gauge aspiration needles. Rigenera (Human Brain Wave, Italy) is a commercial tissue disaggregator able to filter and select progenitor cells with a size of 50 μm from micrograft samples (adipose tissue, dental pulp, periosteum, etc.). Celution is an automated processing unit for the standardized extraction, washing, and concentration of autologous adipose‐derived SVF.

Techniques requiring substantial manipulation are highlighted in gray.

a At 6 weeks (see discussion).

b Alveolar thickness loss.

c At 6 months.

d Described in text but without quantitative outcome measurement.

Abbreviations: ARP, alveolar ridge preservation; A‐SVF, adipose stromal vascular fraction; BMAC, bone marrow aspirate concentrate; BMSCs, bone marrow stem cells; CCT, controlled clinical trial; DPSCs, dental pulp stem cells; GA, general anesthetic; GMP, good manufacturing practice; LA, local anesthetic; LRA, lateral ridge augmentation; mod; moderate; PdlSCs, periodontal ligament stem cells; PdSCs, periosteum derived stem cells; PERIO, periodontal regeneration; RCT, randomized controlled clinical trial; RoB, risk of bias; SC, some concern; SINUS, sinus augmentation; TRCs, tissue repair cells.

### Risk of Bias and Methodological Quality

**Figure 2:**
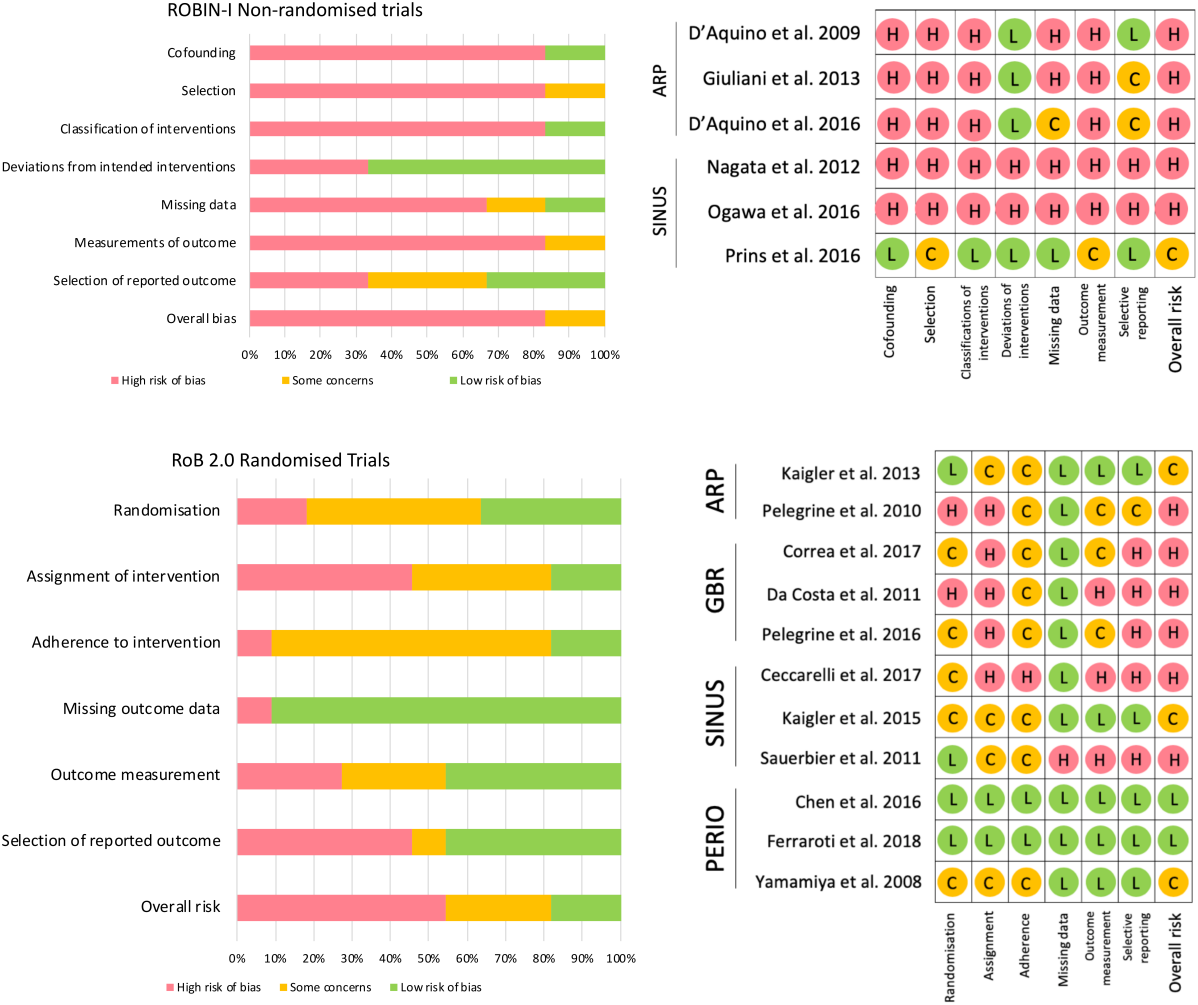
Risk of bias assessment for randomized (RoB 2.0) and nonrandomized (ROBIN‐I) trials. Abbreviation: RoB, risk of bias.

For the ROBIN‐I assessment of nonrandomized clinical trials, five of the six studies included were considered to be at a high risk of bias. Among the RCTs assessed with the Cochrane RoB 2.0 tool, two studies showed a low risk of bias, three exhibited some methods which raised some concerns about the risk of bias, and six reported systematic errors in design, adherence or reporting which translated in a high risk of bias. Figure 2 displays the results of the bias and methodological quality assessment for all studies (for further information, see Supporting Information).

### Narrative Synthesis and Quantitative Analysis

**Figure 3:**
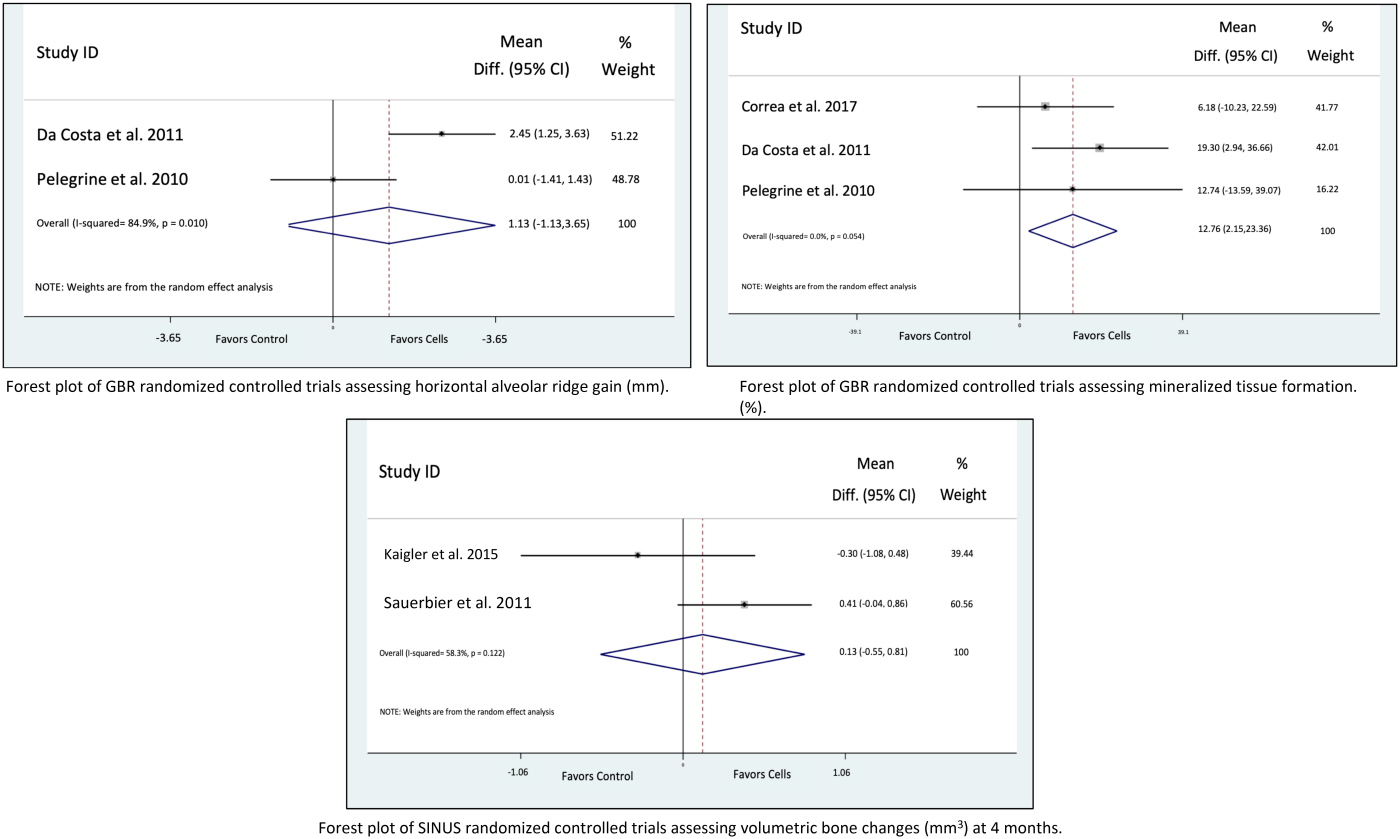
Forest plots of studies assessing alveolar bone regeneration.

**Figure 4:**
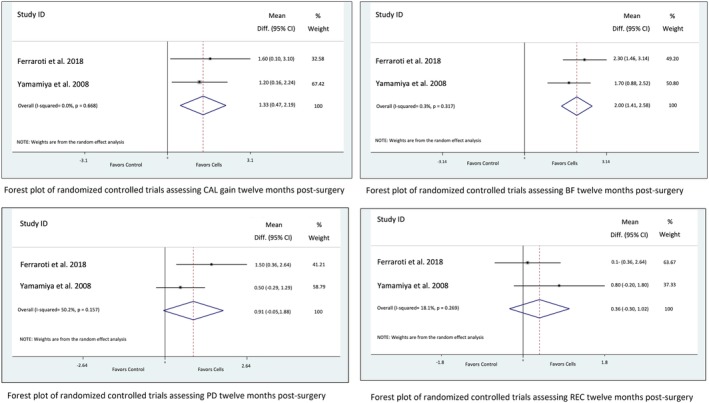
Forest plots of randomized controlled clinical trials assessing clinical outcomes for periodontal regeneration.

The reader is referred to the extensive tables in the Supporting Information for a comprehensive summary of clinical, histological, and other relevant outcome data reported in the included studies categorized according to defect type. A brief synthesis is presented below with Forest plots of all meta‐analysis carried out presented in Figures 3 and 4.

### Alveolar Ridge Preservation

Due to significant differences in study characteristics, it was not possible to complete meta‐analysis of effects size for the studies reporting on the use of stem cells for alveolar ridge preservation; a short narrative synthesis is therefore presented.

Of the four original reports included 14, 15, 16, 17, two were CCTs 14, 15. D'Aquino et al. 14 reported statistically significant improvements in radiographic ridge regeneration (%) 12 months after extraction. Another study by D'Aquino et al. 15 showed that the percentage of resorption of the alveolar ridge was less for the test group than the control group both in the vertical (*Mean diff* 36.5%, *p* < .001) and the horizontal (*Mean diff* 38.33%, *p* < .001) dimensions 45–90 days after ridge preservation surgery. Another two RCTs reported on the use of BMSCs 16, 17. In one of the studies, significant differences in percent of radiographic bone height were shown at 6 weeks (*Mean diff* 23.6% [6.02–41.09], *p* = .01) but the benefit of the cell‐based therapy was lost at 12 weeks (*Mean diff* 5.4% [−12.11, 22.95], *p* = .28) 16. Histological analyses showed that there was a nonstatistically significant increase in the percentage of Bone Tissue Area/Tissue Area at the test sites compared with control at 6 weeks (*Mean diff* 13.9% [−5.03–33.2], *p* = .09) and no difference at 12 weeks (*Mean diff* 0.2% [−19.1% 19.4], *p* = .49) 16. The second RCT reported that at 6 months after tooth extraction, there were no differences between test and control in direct linear measurements of alveolar bone height, but the sites treated with cell therapy showed significantly less horizontal ridge reduction (T: 1.14 mm ± 0.87 mm; C: 2.46 ± 0.4 mm, *p* = .014) with the test group losing on average 13.61% ± 12.5% and the control group 31.35% ± 11.88% of the original ridge width (*p* = .006) 17. The percentage of mineralized bone in the core bone biopsies was similar in both groups (T: 45.47% ± 7.21%, C: 42.87% ± 11.33%, *p* = .36) 17. Both studies reported in the number of cases requiring further bone grafting at the time of implant placement and reported a lesser need for additional grafting procedures in cell‐based treated sites compared with control.

### Guided Bone Regeneration: Lateral Ridge Augmentation

Two studies reporting radiographic horizontal bone width linear measurements outcomes 19, 20 and three 18, 19, 20 studies reporting percentage of newly formed bone histologically were included in separate meta‐analysis in the guided bone regeneration category. Random effects meta‐analysis of the studies reporting alveolar bone width showed a mean extra 1.13 mm alveolar bone gain (95% CI, −1.13, 3.65), 4 to 6 months post‐surgery, favoring cell‐based therapies with considerable heterogeneity (*I* ^2^ = 84.9%). The combined estimate for the three studies reporting data for percentage of mineralized tissue histologically was 12.76% (95% CI, 2.15, 23.36; *I* ^2^ = 0.0%). The three RCTs reported no complications or adverse events.

### Sinus Augmentation

Three CCTs and three RCTs reported data for radiographic changes in alveolar ridge dimension. However due to discrepancies between studies, only two studies 25, 26 evaluating increase in bone volume in the augmented area were pooled for random‐effects meta‐analysis. The studies reported contradicting results and, as a result, a nonsignificant mean volumetric bone gain of 0.13 mm^3^ (95% CI −0.55, 0.81) in favor of the stem cell groups with substantial heterogeneity (*I* ^2^ = 58.3%, *p* = .122) was observed. These data are in line with the outcomes of the other four studies with an adequate control groups 21, 22, 23, 24 showing no differences in alveolar ridge vertical or volumetric gain between cell‐based approaches and positive controls without cell endorsement (see Supporting Information).

Histologically, two studies 23, 26 reported similar findings: there were no significant differences between groups with or without cell therapy for new bone formation. Furthermore, two studies 23, 25 reported a nonsignificant higher bone volume fraction within the newly regenerated bone as assessed by micro‐CT analysis. The publications showed no complications or significant adverse events with the exception of one study that reported one injury of the inferior alveolar nerve when harvesting autogenous bone and two other infections of the bone donor site.

### Periodontal Regeneration

For studies aiming to achieve periodontal regeneration, one of the RCTs did not report clinical measurements beyond 3 months and did not report the standard deviations of the differences in radiographic bone fill at 12 months postop 27. For this reason, two studies 28, 29 assessing CAL, PD, REC, and BF 12 months after surgery were included in separate meta‐analysis. The combined estimate for the two studies showed significant benefits of using cell therapy for periodontal regeneration in terms of CAL (1.33 mm; 95% CI, 0.47, 2.19; *I* ^2^ = 0.0%) and radiographic BF (2 mm; 95% CI, 1.41, 2.58; *I* ^2^ = 0.3%) with no observed heterogeneity and a nonsignificant improvement in PD (0.91 mm; 95% CI, −0.05, 1.88; *I* ^2^ = 50.2%), and REC (0.36 mm; 95% CI, −0.30, 1.02; *I* ^2^ = 18.1%). This positive effect is further illustrated by one of the studies reporting CAL gain as a % of the original defect, showing 83.5% (±31.7) defect resolution in cell‐grafted sites compared with 55.0% (±21.9) in control sites 29. Finally, the other study included in the meta‐analysis demonstrated a significantly higher percentage of sites showing CAL gain >4 mm (73.3% vs. 28.6%) and less sites with probing pocket depths >6 mm (0% vs. 14.3%) for the defects treated with cell techniques 28.

## Interpretation

### Overall Quality, Strength, and Consistency of Evidence

The low number of included studies does not come as a surprise given that only recently the application of cell‐based therapies for regeneration of alveolar bone and periodontal tissues has been reported. We focused our research question around RCTs and CCTs which could contribute clinical outcome measures and not only histology analysis. For this reason, the secondary histology outcomes reported in this review were limited due to many studies reporting only histology analyses being excluded.

The CCTs included in this review were considered to be at a high risk of bias 14, 15, 21, 22, 23. On the other hand, the RCTs comprised a somehow less biased data set with 5 of the 11 original reports showing low levels of bias 16, 25, 27, 28, 29. Furthermore, there was a great degree of variability on study design as well as many different cell populations and scaffold combinations, making the test and control groups not homogenous as a whole. The overall estimates from the meta‐analysis, albeit they represent the best‐available evidence, should be interpreted with caution.

### Clinical Applicability: Beyond the Hospital/University Setting

There were virtually no complications and no severe adverse events across all of the included studies in all the different defect categories, demonstrating the safety of this treatment modality. Although there were insufficient data to draw any conclusions regarding best cell‐based treatment strategies, an appraisal of the clinical applicability of the different techniques identified through the systematic search is warranted. Our assessment of the different techniques will appraise aspects which may be relevant for widespread adoption later on the translational pathway. We hope this will help identifying techniques which may be prioritized in future research.

The studies 14, 15, 16, 17 assessing changes after grafting of the alveolar ridge following tooth extraction suggest that ARP should be a targeted intervention to demonstrate the clinical potential of cell‐based therapy for alveolar bone regeneration. The clinical strategy after a tooth is extracted most often involves the decision to place a dental implant with a type 1/type 2 placement 33 leaving the ridge to heal undisturbed. The placement of slow‐resorbing bone substitutes unnecessarily lengthens the waiting period before a dental implant can be inserted 9. In the current review, limited data suggests that significant improvements may be obtained on reducing ridge resorption and improving bone quality within the first 6 weeks of healing when progenitor cells are endorsed in fast‐resorbing collagen sponges and grafted on “extraction socket” defects. The additional benefit provided by cell therapy may justify the associated costs of (some of) the techniques by reducing the need of major grafting while shortening treatment times. Similar conclusions may be drawn for lateral ridge augmentation of large horizontal defects. On the other hand, sinus augmentation procedures seem less appropriate for this technique 21, 22, 23, 24, 25, 26. A marginal or no effect was reported both for clinical and histology outcomes. This is perhaps related to the fact that the maxillary sinus defect is, by nature, a spontaneous‐healing model 34. Although excellent outcomes were shown in the periodontal regeneration studies, the outcomes in the control groups cannot be considered comparable to what is expected from gold standard therapy according to previous research. Therefore, further research comparing this technique to Guided Tissue Regeneration or well‐proven bioactive agents such as Emdogain® is needed to elucidate whether there is merit on pursuing this application further. Both in sinus augmentation surgery and periodontal regeneration, it is evident that a wide range of techniques have proven to be largely successful on achieving predictable and highly successful outcomes, and, hence, it may be difficult to ever find a large enough “perceived clinical benefit” which could justify the cost and operational needs of cell‐based therapies. An exception may be the use of cost‐effective “minimal” manipulation techniques such as the one described by Ferraroti et al. 28

The regeneration of tissues depends mainly on three key players: scaffolds, cells, and biomolecules coming together to orchestrate a series of spatial‐temporal events which result in a pattern of healing that resembles the original components, structure, and function of the lost tissues. There were significant differences between the techniques appraised in this review on the use of scaffolds and “biologics.” For instance, there was a wide range of different scaffolds which were used as “carriers” for the cells. When the studies are stratified according to defect type, it becomes apparent that the scaffolds were selected according to the biomechanical and biological requirements of the defects which were being reconstructed and not for their ability to improve cell attachment and/or behavior. For this reason, we will focus the discussion on the cell harvesting and manipulation steps.

We based our “clinical applicability” appraisal on domains like those described in Roger's diffusion of innovation theory 35 (see Table 1). This theory has been previously tailored and implemented for analysis of key elements influencing the widespread adoption of new technologies/techniques in healthcare 36 and dentistry 37. Stem cells can be categorized in four different groups depending on their source 38: (a) embryonic tissues, (b) fetal tissues, (c) postnatal tissues, and (d) induced pluripotent stem (iPS) cells. Embryonic stem cells have the highest proliferative rate and are able to differentiate into any of the three embryonic germ layers (ectoderm, mesoderm, and endoderm) and can therefore be a source for almost all cell types within the human body. However, their consideration for use is affected by ethical issues since their isolation involves the use of human embryos 38. In the future, the use of iPS cells 39 would circumvent these ethical concerns. Another alternative currently available is the recruitment of postnatal stem cells, more widely known as MSCs derived broadly from nearly every organ and tissue. Compared with embryonic stem cells and iPS cells, MSCs have restricted differentiation potential and proliferative rates, showing great variability depending on the tissue source 38. Although the term stem cell therapy is widely used to describe the use of any of the cell populations above or even to also include therapies using cells further committed in the differentiation pathway, it seems more appropriate to use more focused terms which provide a better description of the cell population being applied providing a better description of their differentiation potential (i.e., tissue‐specific cell therapy). We identified therapies involving five distinct cell populations: BMSCs obtained from BMA 16, 17, 18, 19, 20, 26, DPSCs from the dental pulp of extracted teeth 14, 28, PdSCs 15, 21, 22, 24, 29 from gingival connective tissue samples, PdlSCs 27 from extracted teeth, and A‐SVF 23 from adipose tissue.

We consider the invasiveness of the harvesting procedure as one of the factors potentially affecting adoption. The techniques involving DPSCs and PdlSCs harvesting require the extraction of a tooth. This invasive procedure would only be appropriate in cases were extractions are already scheduled, limiting its applicability. The harvesting of A‐SVF requires a liposuction under general anesthesia and is therefore considered highly invasive. Bone marrow aspirates were carried out under local anesthesia and, in some cases, conscious sedation and were rated as moderately invasive. The less invasive technique was the harvesting of gingival connective tissue from intra‐oral sources. The clear advantage of this technique is that tissues are readily available. Besides their invasiveness, the need of a BMA or liposuction adds an additional layer of complexity due to the need to contact and organize specialists outside the dental office while also adding significant costs.

After harvesting, the cells were either “minimally” or “substantially” modified before application in the surgical site. The rationale for cell expansion is based on the fact that only a small fraction of the cells within the donor tissues are characterized as MSCs. Billions of cells can be generated from as little as 1 ml of bone marrow aspirate following ex vivo expansion 38, 40. On the other hand, despite low cell yields, “minimal manipulation” of the source tissue will include cells and matrix proteins other than MSCs which may also play a key role for tissue regeneration. Furthermore, there is not known threshold above or below in which cell yield is relevant for improving clinical outcomes. It has recently been suggested that we should “move away from autograft‐based therapy” and that “the preferred therapeutic strategy moving forward is the use of ex vivo expanded MSC preparations rather than whole bone marrow aspirate transplantation or autografts that contain limited MSC numbers diluted within a heterogeneous population of blood/ immune cells 38.” ex vivo cell expansion is a complex process that requires significant investment in terms of time, resources, and capital, and there is currently no evidence to support an additional clinical benefit compared with “minimal” manipulation cell therapies, at least for the management of the alveolar bone defects most commonly found in day to day practice. It is also evident, albeit only from the limited number of clinical trials available, that techniques requiring “minimal” manipulation of tissues and cells have shown clinical and histology benefits in all the defect models reviewed, casting doubts on the “absolute need” for cell expansion. Furthermore, cell survival in vivo following transplantation may be short‐lived 41. It has been suggested that MSCs effects go beyond their own innate ability to survive and differentiate into tissue forming cells but lies within their ability to “secrete bioactive factors that are immunomodulatory and trophic 42.” The use of cellular therapies may act in the clinical context by one of either two broad mechanisms: (a) Cell Replacement—whereby transplanted (generally autologous cells) successfully are accepted to the local host site and then begin the process of tissue regeneration of the local defect; and/or (b) Cell Empowerment—mechanism whereby cells (either autologous or allogeneic) display a more transient effect in the local area to release factors that may promote the regenerative response 43. Whether or not ex vivo cell expansion is a prerequisite for the success of cell‐based therapies is an important question, one should be answered through future well‐designed randomized controlled clinical trials.

The time that lapsed from cell harvesting to application in “substantially manipulated” therapies ranged from 12 days to over 6 weeks 14, 16, 21, 22, 25, 27, 29. Furthermore, cell expansion requires GMP grade laboratories and personnel. A publication of a report of three cases using these techniques showed that the GMP level laboratory preparatory costs to the Finnish healthcare system were the equivalent of £8,900 per patient 44. In addition to these elevated expenses, the need of significant external resources and the high complexity of the interventions make these therapies impractical outside the hospital/university settings. Among the “minimally manipulated” techniques, A‐SVF obtained through processing of adipose samples using an automated processing unit (Celution®, Cytori Therapeutics, USA) is the most invasive, expensive, and complex alternative followed by the use of BMAC obtained through processing with a commercially available system (Harvest Terumo® BCT, Terumo Medical do Brasil, Brazil) after iliac crest aspiration. A viable alternative may be the use of a commercially available tissue disaggregator (Rigenera®, Human Brain Wave, Italy) able to filter progenitor cells with a size of 50 μm from micrograft tissue samples (i.e., adipose tissue, dental pulp, periosteum) 45. The Rigenera system presented clinical data in four studies investigating all defect models covered in this review. Using this device, DPSCs and PdSCs were obtained from dental pulp tissue and gingival connective tissue samples showing promising results.

### Outstanding Questions

Based on a limited number of studies, cell‐based therapies have the potential to improve outcomes of regenerative treatment for the reconstruction of alveolar bone and periodontal tissues. We identified extraction socket defects and challenging lateral and vertical bone defects requiring bone block grafts as ideal candidates for the adjuvant application of MSCs. This review showed that there is insufficient evidence to identify best‐performing treatment modalities among the different cell‐based techniques described in the included publications. This research synthesis has also highlighted a high risk of bias in most of the studies and the need for well‐designed randomized clinical controlled trials including the gold standard of treatment as a positive control. Given the complexity, invasiveness and costs associated with techniques which include “substantial manipulation” of tissues and cells, their clinical benefit when compared with “minimal manipulation” must be elucidated in future trials. Further research evaluating the clinical effect and cost‐effectiveness of simple, fast, and economical methods for cell harvesting and processing is warranted.

## Author Contributions

F.M.S.: protocol design, publication screening, data abstraction, data analysis, interpretation of results, writing of the manuscript; Y.L.: protocol design, publication screening, data abstraction, data analysis, interpretation of results, reviewing the manuscript; M.O., J.B.: protocol design, data analysis, interpretation of results, review of the manuscript; W.V.G., F.D.: interpretation of results, review of the manuscript.

## Disclosure of Potential Conflicts of Interest

The authors indicated no potential conflicts of interest. UCL and/or UCLH and UCLH CBRC paid salaries as part of their research/clinical roles to all authors and researchers involved in the project.

## Supporting information


**Appendix** S1: Supporting Information 1

## Data Availability

The data that support the findings of this study are available on request from the corresponding author. The data are not publicly available due to privacy or ethical restrictions.
