# Supplementary material for: Cell‐Based Therapies for Alveolar Bone and Periodontal Regeneration: Concise Review
Source: Stem Cells Transl Med. 2019 Nov 6;8(12):1286–95. doi: 10.1002/sctm.19-0183 (PMC6877771; doi:10.1002/sctm.19-0183)
Supplement: Supplementary file 1 — Appendix S1: Supporting Information 1 [file SCT3-8-1286-s001.pdf]

Electronic search strategies

MEDLINE

1. exp Alveolar Bone Loss/su [Surgery]
2. exp Alveolar Ridge Augmentation/
3. exp Tooth Extraction/ or exp Tooth Socket/
4. exp Guided Tissue Regeneration, Periodontal/ or exp Periodontal Ligament/ or exp Bone Regeneration/ or exp Regeneration/
5. exp Sinus Floor Augmentation/
6. exp Tissue Engineering/mt [Methods]
7. (mesenchymal or stem or stromal or MSC*).mp. [mp=title, abstract, original title, name of substance word, subject heading word, floating sub-heading word, keyword heading word, protocol supplementary concept word, rare disease supplementary concept word, unique identifier, synonyms]
8. cell therapy.mp. [mp=title, abstract, original title, name of substance word, subject heading word, floating sub-heading word, keyword heading word, protocol supplementary concept word, rare disease supplementary concept word, unique identifier, synonyms]
9. "Cell- and Tissue-Based Therapy"/
10. 1 or 2 or 3 or 4 or 5
11. 6 or 7 or 8 or 9
12. 10 and 11
13. Epidemiologic Studies/
14. Cohort Studies/
15. Follow-Up Studies/
16. Longitudinal Studies/
17. Prospective Studies/
18. (cohort adj (study or studies)).tw.
19. cohort analy*.tw.
20. (follow up adj (study or studies)).tw.
21. (observational adj (study or studies)).tw.
22. (longitudinal adj (study or studies)).tw.
23. (prospective adj (study or studies)).tw.
24. or/13-23
25. 12 and 24

EMBASE

1. exp periodontitis/su [Surgery]
2. exp alveolar bone loss/su [Surgery]
3. exp alveolar ridge augmentation/
4. exp oral surgery/
5. Guided Tissue Regeneration, Periodontal/ or exp Periodontal Ligament/ or exp Bone Regeneration/ or exp Regeneration/
6. exp Sinus Floor Augmentation/
7. tissue engineering/
8. (mesenchymal or stem or stromal or MSC*).mp. [mp=title, abstract, heading word, drug trade name, original title, device manufacturer, drug manufacturer, device trade name, keyword, floating subheading word, candidate term word]
9. cell therapy.mp. [mp=title, abstract, heading word, drug trade name, original title, device manufacturer, drug manufacturer, device trade name, keyword, floating subheading word, candidate term word]
10. "Cell- and Tissue-Based Therapy"/
11. 1 or 2 or 3 or 4 or 5 or 6

12. 7 or 8 or 9 or 10
13. 11 and 12
14. exp cohort analysis/
15. exp follow up/
16. exp longitudinal study/
17. exp prospective study/
18. (cohort adj (study or studies)).tw.
19. cohort analy*.tw.
20. (observational adj (study or studies)).tw.
21. (longitudinal adj (study or studies)).tw.
22. (prospective adj (study or studies)).tw.
23. (epidemiologic* adj (study or studies)).tw.
24. or/14-23
25. 13 and 24

LILACS
(Periodontitis OR Alveolar Bone Loss OR Periodontal Surgery OR Oral surgery OR Tooth Extraction OR Guided Tissue Regeneration OR Periodontal Regeneration OR Sinus Augmentation OR Bone Regeneration) and (Epidemiologic Studies OR Cohort Studies OR Follow-Up Studies OR Longitudinal Studies OR Prospective Studies) [Subject Descriptor]
or
(Periodont$ or (gingiva$ and pocket$) or Surg$ and ((cohort and (study or studies)) or cohort analy$ or (follow up and (study or studies)) or (observational and (study or studies)) or (longitudinal and (study or studies)) or (prospective and (study or studies))) [Words]

OpenGrey
(Periodont* OR (gingiva* AND pocket*)) AND Surg* AND((cohort AND (study OR studies)) OR "cohort analy*" OR ("follow up" AND (study OR studies)) OR (observational AND (study OR studies)) OR (longitudinal AND (study OR studies)) OR (prospective AND (study OR studies)) OR (epidemiologic* AND (study or studies)))

Journals for Hand searching

- Bone,
- Cell Transplantation,
- Clinical Advances in Periodontics,
- Clinical Oral Implants Research,
- Implant Dentistry,
- International Journal of Biomaterials,
- International Journal of Periodontics & Restorative Dentistry,
- International Journal of Oral and Maxillofacial Surgery,
- Journal of Clinical Periodontology,
- Journal of Dental Research
- Journal of Oral and Maxillofacial Surgery,
- Journal of Periodontology,
- Journal of Periodontal Research,
- Oral & Maxillofacial Surgery,
- Regenerative Therapy,
- Stem Cells,
- Stem Cell Research & Therapy,
- Stem Cells Translational Medicine.

Methods

Study selection

Titles and abstracts of the studies identified in the searches were screened by two of the review authors (FM and YL) in duplicate and independently. Subsequently, the full text of all publications appearing to meet the inclusion criteria or for which there was not sufficient information in the title and abstract to make a decision, were obtained. At this first stage, any study considered as potentially relevant by at least one of the reviewers was included for the next screening phase. Subsequently, the full-text publications were evaluated in duplicate and independently by the same review examiners. The examiners were calibrated with the first 5 full text consecutive publications. Any disagreement on the eligibility of studies was resolved through discussion between both reviewers until consensus was reached, or through arbitration by a third reviewer if needed (FDA). All potentially relevant studies that did not meet the eligibility criteria were excluded and the reasons for exclusion recorded. Inter-examiner agreement following full-text assessment was calculated via Kappa statistics.

Study details were collected using previously agreed forms specifically designed for data extraction in this review and which were firstly piloted in a small number of studies. Two of the review authors (FM and YL) extracted all relevant data from all included studies independently. Any disagreements were resolved through debate and consensus or, ultimately, through assessment of a third reviewer (FDA). When a publication indicated the measurement of the primary outcome, but this was not reported/unclear, the authors were contacted in an attempt to obtain the missing data.

Determination of outcome measures

a) ARP: bone dimensional changes occurring in the ridge walls following tooth extraction and the socket preservation therapy, reported as linear measurements and/or volumetric changes.

b) GBR/SINUS: defect size pre- and postoperatively and/or the amount of augmentation reported as linear measurements and/or volumetric changes.

c) PERIO: Clinical Attachment Level (CAL) change (or variants), all probing methods were included. Radiographic bone fill.

Secondary outcome measures for alveolar bone augmentation studies included:

- Histologically, the formation of bone tissue following the use of cell-based therapies as evaluated by histomorphometry (% new bone formation, % of residual graft material present).
- Soft tissue volumetric changes, the presence and amount of keratinized tissue at time of implant placement (yes/no and mm), the feasibility of implant placement given bone dimensions (yes or no), the need for soft and/or hard tissue augmentation at the time of implant placement (frequency and technique) and patient-reported outcome measures.
- Complication rates.

For periodontal regeneration studies, if available:

- Histologically, the nature of healing associated with defect resolution would be recorded (i.e. true regeneration vs repair) as well as histomorphometry outcomes (e.g. % of new periodontal attachment).
- Other relevant clinical outcomes including probing pocket depth and recession.

Data extraction and analysis

Study details were collected using previously agreed form(s) specifically designed for data extraction in this review and which was firstly piloted in the first 5 consecutive full text studies included in the review. Two of the review authors (FM and YL) independently extracted all relevant data from all included studies. Any disagreements were resolved through debate and consensus or through assessment of a third reviewer (FDA).

Quality assessment

Risk of bias for randomised controlled clinical trials was assessed using the Revised Cochrane risk-of-bias tool for randomized trials. For non-randomised trials the Risk Of Bias In Non-randomized Studies – of Interventions (ROBINS-I) assessment tool was used.

Data synthesis and analysis

Data synthesis including included both descriptive and quantitative methods. Data was first entered into evidence tables stratified by defect model and study design. Studies were stratified according to defect model and study design in the evidence tables. If feasible, meta-analysis of eligible studies was completed. Decisions on which studies to include for meta-analysis was made depending on similarity of study characteristics related to each outcome measure and the main research question.

The chi-square test and the I2 measures were used to assess statistical heterogeneity of relevant outcomes amongst included studies. Although using thresholds to assess heterogeneity may be misleading, we followed guidance from the Cochrane Handbook to interpret I2.

An overall estimate of the mean effect size of relevant outcomes, with its 95% confidence interval, was obtained through meta-analysis when it was deemed appropriate. A random effects model-approach was taken if there was evidence of statistical heterogeneity while a fixed effects model was used if there was no heterogeneity. The data collected from the studies was limited for quantitative analysis (meta-analysis), resulting only in a few exploratory assessments. Statistical analyses were conducted by FM using Stata (StataCorp. 2018. Stata Statistical Software: Release 15. College Station, TX: StataCorp LP).

Excluded full-text studies.

Study ID	Reason for exclusion
Alveolar ridge preservation	
Graziano et al. 2013	Case report
Kaigler et al. 2010	Case report
Lorenz et al. 2018	Does not use stem cell therapy
Sheth et al. 2017	Abstract only – no full text available
Lateral/Vertical ridge augmentation	
Abrahamsson et al. 2012	Test group does not include cell-based approach
Cortellini et al. 2018	Does not use stem cell therapy
Kulakov et al. 2008	Case series – no control group
Rajan et al. 2014	Case report
Sauerbier et al. 2013	Case report
Soltan et al. 2007	Case series
Soltan et al. 2010	Case series
Yamada et al. 2013	Case report
Sinus augmentation	
Beaumont et al. 2008	Case series - no control group
Brunelli et al et al. 2013	Case report
Fuerst et al. 2009	No control group
Gonshor et al. 2011	Does not record/report primary outcome
Lupi et al. 2018	Case report
McAllister et al. 2009	Case series
Pasquali et al. 2016	Does not record/report primary outcome
Pohl et al. 2016	Case series – no control group
Rickert et al. 2011	Does not record/report primary outcome
Rodriguez Y et al. 2017	Does not record/report primary outcome
Sauerbier et al. 2010	Does not record/report primary outcome
Schmelzeisen et al. 2011	Case report
Schimming et al. 2004	Case series – no control group
Shayesteh et al. 2008	Case series – no control group
Smiler et al. 2007	Case series
Springer et al. 2006	Case series
Voss et al. 2010	Does not record/report primary outcome
Yamada et al. 2008	Case series
Zizelmann et al. 2007	Does not record/report primary outcome
Combination	
Cerruti et al. 2007	Case series – no control group
Katagiri et al. 2016	Case series – no control group
Montesani et al. 2011	Case report
Smiler et al. 2004	Case series
Trautvetter et al. 2011	Case series

Ueda et al. 2005	Case series
Ueda et al. 2008	Case series
Yamada et al. 2013	Case series
Periodontal regeneration	
Aimetti et al. 2015	Case series - no control group
Aimetti et al. 2018	Case series - no control group
Baba et al. 2016	Control group did not receive surgical treatment
Chen et al. 2012	Review with case report
Feng et al. 2010	Case series – no control group
Hou et al. 2003	Case series – no control group
Iwata et al. 2018	Case series – no control group
Li et al. 2016	Case series – no control group
McAllister 2011	Case report
Okuda et al. 2009	Case series
Okuda et al. 2013 (Follow-up)	Case series
Sankaranarayananet al. 2013	Case report
Santana et al. 2009	Does not use stem cell therapy
Yamada et al. 2006	Case report
Other defects	
Colangeli et al. 2017	Case series, defect model does not fulfil inclusion criteria
Kontio et al. 2012	Case report, defect model does not fulfil inclusion criteria
Pena Gonzalez et al. 2016	Pre-clinical defect model

Excluded full-text studies: references.

1. Graziano A, Carinci F, Scolaro S, D'Aquino R (2013) Periodontal tissue generation using autologous dental ligament micro-grafts: Case report with 6 months follow-up. Ann Oral Maxillofac Surg 1:20.
2. Kaigler, Darnell; Pagni, Giorgio; Park, Chan-Ho; Tarle, Susan A; Bartel, Ronnda L; Giannobile, William V. (2010) Angiogenic and osteogenic potential of bone repair cells for craniofacial regeneration. Tissue engineering. Part A.; 16(9):2809-20.
3. Lorenz, J., et al. (2018). "Injectable Bone Substitute Material on the Basis of ß-TCP and Hyaluronan Achieves Complete Bone Regeneration While Undergoing Nearly Complete Degradation." Int J Oral Maxillofac Implants 33(3): 636-644-636-644.
4. Sheth, N., et al. (2017). "Human mandible bone defect repair by the grafting of dental pulp stem/progenitor cells-a pilot study." International journal of oral and maxillofacial surgery 46(Supplement 1): 215.
5. Abrahamsson, P., et al. (2012). "Periosteal expansion before local bone reconstruction using a new technique for measuring soft tissue profile stability: a clinical study." Journal of oral and maxillofacial surgery : official journal of the American Association of Oral and Maxillofacial Surgeons 70(10): e521-530.
6. Cortellini, S., et al. (2018). "Leucocyte- and platelet-rich fibrin block for bone augmentation procedure: A proof-of-concept study." J Clin Periodontol 45(5): 624-634.
7. Kulakov AA; Goldshtein DV; Grigoryan AS; Rzhaninova AA; Alekseeva IS; Arutyunyan IV; Volkov AV. (2008) Clinical study of the efficiency of combined cell transplant on the basis of multipotent mesenchymal stromal adipose tissue cells in patients with pronounced deficit of the maxillary and mandibulary bone tissue. Bulletin of Experimental Biology & Medicine; 146(4):522-5.
8. Rajan, A., et al. (2014). "Optimized cell survival and seeding efficiency for craniofacial tissue engineering using clinical stem cell therapy." Stem cells translational medicine 3(12): 1495-1503.

9. Sauerbier, Sebastian; Giessenhagen, Bernd; Gutwerk, Wolfgang; Rauch, Petra; Xavier, Samuel P; Oshima, Toshiyuki; Nagursky, Heiner; Gutwald, Ralf; Schmelzeisen, Rainer. (2013) Bone marrow aspirate concentrate used with bovine bone mineral to reconstruct vertical and horizontal mandibular defects: report of two techniques. International Journal of Oral & Maxillofacial Implants; 28(5):e310-4.
10. Soltan, Muna; Smiler, Dennis; Prasad, Hari S; Rohrer, Michael D. (2007) Bone block allograft impregnated with bone marrow aspirate. Implant Dentistry; 16(4):329-39.
11. Soltan, Muna; Smiler, Dennis; Soltan, Christine; Prasad, Hari S; Rohrer, Michael D (2010) Bone grafting by means of a tunnel dissection: predictable results using stem cells and matrix. Implant Dentistry; 19(4):280-7.
12. Yamada Y; Nakamura S; Ito K; Umemura E; Hara K; Nagasaka T; Abe A; Baba S; Furuichi Y; Izumi Y; Klein OD; Wakabayashi T. (2013) Injectable bone tissue engineering using expanded mesenchymal stem cells. Stem Cells; 31(3):572-80.
13. Beaumont C, Schmidt RJ, Tatakis DN, Zafiropoulos GG. (2008) Use of engineered bone for sinus augmentation. J Periodontol ;79(3):541-8.
14. Brunelli, G., et al. (2013). "Sinus lift tissue engineering using autologous pulp micro-grafts: A case report of bone density evaluation." J Indian Soc Periodontol 17(5): 644-647.
15. Fuerst, G., et al. (2009). "Are culture-expanded autogenous bone cells a clinically reliable option for sinus grafting?" Clinical oral implants research 20(2): 135-139.
16. Gonshor, A., et al. (2011). "Histologic and histomorphometric evaluation of an allograft stem cell-based matrix sinus augmentation procedure." The International journal of oral & maxillofacial implants 26(1): 123-131.
17. Lupi SM; Rodriguez Y Baena A; Todaro C; Ceccarelli G; Rodriguez Y Baena R. (2018) Maxillary Sinus Lift Using Autologous Periosteal Micrografts: A New Regenerative Approach and a Case Report of a 3-Year Follow-Up. Case Reports in Dentistry; 2018:3023096.
18. McAllister BS; Haghighat K; Gonshor A. (2009) Histologic evaluation of a stem cell-based sinus-augmentation procedure. Journal of Periodontology; 80(4):679-86.
19. Pasquali, P. J. & Aloise, A. C. (2016) Can bone marrow aspirate concentrate change the mineralization pattern of the anterior maxilla treated with xenografts? A preliminary study. Contemp Clin Dent 7, 21-26.Pohl et al. 2016
20. Pohl, V., et al. (2016). "A New Method Using Autogenous Impacted Third Molars for Sinus Augmentation to Enhance Implant Treatment: Case Series with Preliminary Results of an Open, Prospective Longitudinal Study." Int J Oral Maxillofac Implants 31(3): 622-630.
21. Rodriguez, Y. B. R., et al. (2017). "Autologous Periosteum-Derived Micrografts and PLGA/HA Enhance the Bone Formation in Sinus Lift Augmentation." Front Cell Dev Biol 5: 87.
22. Rickert, D., et al. (2011). "Maxillary sinus floor elevation with bovine bone mineral combined with either autogenous bone or autogenous stem cells: a Rodriguez Y et al. 2017
23. Sauerbier, Sebastian; Stricker, Andres; Kuschnierz, Jens; Buhler, Felicia; Oshima, Toshiyuki; Xavier, Samuel Porfirio; Schmelzeisen, Rainer; Gutwald, Ralf. (2010) In vivo comparison of hard tissue regeneration with human mesenchymal stem cells processed with either the FICOLL method or the BMAC method. Tissue Engineering - Part C: Methods; 16(2):215-23.
24. Schmelzeisen R; Gutwald R; Oshima T; Nagursky H; Vogeler M; Sauerbier S. (2011) Making bone II: maxillary sinus augmentation with mononuclear cells--case report with a new clinical method. British Journal of Oral & Maxillofacial Surgery; 49(6):480-2.
25. Schimming, Ronald; Schmelzeisen, Rainer. (2004) Tissue-engineered bone for maxillary sinus augmentation. Journal of Oral & Maxillofacial Surgery; 62(6):724-9.
26. Shayesteh, Y. S., et al. (2008). "Sinus augmentation using human mesenchymal stem cells loaded into a beta-tricalcium phosphate/hydroxyapatite scaffold." Oral surgery, oral medicine, oral pathology, oral radiology, and endodontics 106(2): 203-209.
27. Smiler, Dennis; Soltan, Muna; Lee, Joseph W. (2007) A histomorphogenic analysis of bone grafts augmented with adult stem cells. Implant Dentistry; 16(1):42-53.
28. Springer, I. N., et al. (2006). "Two techniques for the preparation of cell-scaffold constructs suitable for sinus augmentation: steps into clinical application." Tissue engineering 12(9): 2649-2656.

29. Voss, P., et al. (2010). "Bone regeneration in sinus lifts: comparing tissue-engineered bone and iliac bone." British Journal of Oral and Maxillofacial Surgery 48(2): 121-126.
30. Yamamiya, K., et al. (2008). "Tissue-engineered cultured periosteum used with platelet-rich plasma and hydroxyapatite in treating human osseous defects." Journal of periodontology 79(5): 811-818.
31. Zizelmann, C., et al. (2007). "Bone formation after sinus augmentation with engineered bone." Clinical oral implants research 18(1): 69-73.
32. Cerruti, Humberto; Kerkis, Irina; Kerkis, Alexandre; Tatsui, Nelson Hidekazu; da Costa Neves, Adriana; Bueno, Daniela Franco; da Silva, Marcelo Cavenaghi Pereira (2007) Allogenous bone grafts improved by bone marrow stem cells and platelet growth factors: clinical case reports. Artificial Organs; 31(4):268-73.
33. Katagiri, W., et al. (2012). "Preliminary and first-in-human clinical study of novel bone regenerative medicine using the conditioned media from bone marrow derived mesenchymal stem cells." Journal of tissue engineering and regenerative medicine 6(SUPPL. 1): 283-284.
34. Montesani, L., et al. (2011). "Sinus augmentation in two patients with severe posterior maxillary height atrophy using tissue-engineered bone derived from autologous bone cells: a case report." The International journal of periodontics & restorative dentistry 31(4): 391-399.
35. Smiler, Dennis; Soltan, Muna. (2004) Bone marrow aspiration: technique, grafts, and reports. Implant Dentistry; 15(3):229-35.
36. Trautvetter, W., et al. (2011). "Tissue-engineered polymer-based periosteal bone grafts for maxillary sinus augmentation: Five-year clinical results." Journal of Oral and Maxillofacial Surgery 69(11): 2753-2762.
37. Ueda, Minoru; Yamada, Yoichi; Ozawa, Ryotaro; Okazaki, Yasuhiro. (2005) Clinical case reports of injectable tissue-engineered bone for alveolar augmentation with simultaneous implant placement. International Journal of Periodontics & Restorative Dentistry; 25(2):129-37.
38. Ueda, Minoru; Yamada, Yoichi; Kagami, Hideaki; Hibi, Hideharu. (2008) Injectable bone applied for ridge augmentation and dental implant placement: human progress study. Implant Dentistry; 17(1):82-90.
39. Yamada Y; Nakamura S; Ito K; Umemura E; Hara K; Nagasaka T; Abe A; Baba S; Furuichi Y; Izumi Y; Klein OD; Wakabayashi T. (2013) Injectable bone tissue engineering using expanded mesenchymal stem cells. Stem Cells; 31(3):572-80.
40. Aimetti M, Ferrarotti F, Mariani GM, Cricenti L, Romano F. (2015) Use of Dental Pulp Stem Cells/Collagen Sponge Biocomplex in the Treatment of Non-Contained Intrabony Defects: A Case Series. J Perio; 5(2): 104-109.
41. Aimetti, M., Ferrarotti, F., Gamba, M. N., Giraudi, M., & Romano, F. (2018). Regenerative treatment of periodontal intrabony defects using dental pulp stem cells: 1-year follow-up case series. The International Journal of Periodontics and Restorative Dentistry, 38, 51–58.
42. Baba S; Yamada Y; Komuro A; Yotsui Y; Umeda M; Shimuzutani K; Nakamura S. (2016) Phase I/II Trial of Autologous Bone Marrow Stem Cell Transplantation with a Three-Dimensional Woven-Fabric Scaffold for Periodontitis. Stem Cells International; 2016:6205910.
43. Chen, F. M., Sun, H. H., Lu, H. & Yu, Q. (2012) Stem cell-delivery therapeutics for periodontal tissue regeneration. Biomaterials 33, 6320-6344.
44. Feng, F; Akiyama, K; Liu, Y; Yamaza, T; Wang, T-M; Chen, J-H; Wang, B B; Huang, G T-J; Wang, S; Shi, S. (2010) Utility of PDL progenitors for in vivo tissue regeneration: a report of 3 cases. Oral Diseases; 16(1):20-8.
45. Hou, L-T; Tsai, Alex Yi-Min; Liu, C-M; Feng, F. (2003) Autologous transplantation of gingival fibroblast-like cells and a hydroxylapatite complex graft in the treatment of periodontal osseous defects: cell cultivation and long-term report of cases. Cell Transplantation; 12(7):787-97.
46. Iwata, T., et al. (2018). "Periodontal regeneration with autologous periodontal ligament-derived cell sheets - A safety and efficacy study in ten patients." Regenerative Therapy 9: 38-44.
47. Li, Y., et al. (2016). "Repair of human periodontal bone defects by autologous grafting stem cells derived from inflammatory dental pulp tissues." Stem Cell Res Ther 7(1): 141-141.

48. McAllister, B. S. (2011). "Stem cell-containing allograft matrix enhances periodontal regeneration: case presentations." The International journal of periodontics & restorative dentistry 31(2): 149-155.
49. Okuda, K., et al. (2013). "Tissue-engineered cultured periosteum sheet application to treat infrabony defects: case series and 5-year results." The International journal of periodontics & restorative dentistry 33(3): 281-287.
50. Okuda, K., et al. (2009). "Treatment of human infrabony periodontal defects by grafting human cultured periosteum sheets combined with platelet-rich plasma and porous hydroxyapatite granules: case series." Journal of the International Academy of Periodontology 11(3): 206-213.
51. Sankaranarayanan, S., et al. (2013). "Periodontal regeneration by autologous bone marrow mononuclear cells embedded in a novel thermo reversible gelation polymer." Journal of stem cells 8(2): 99-103.
52. Santana, Ronaldo B; de Mattos, Carolina Miller Leite; Van Dyke, Thomas. (2009) Efficacy of combined regenerative treatments in human mandibular class II furcation defects. Journal of Periodontology; 80(11):1756-6.
53. Yamada Y; Ueda M; Hibi H; Baba S. (2006) A novel approach to periodontal tissue regeneration with mesenchymal stem cells and platelet-rich plasma using tissue engineering technology: A clinical case report. International Journal of Periodontics & Restorative Dentistry; 26(4):363-9.
54. Colangeli, W., et al. (2018). "Jaw bones regeneration using mesenchymal stem cells. A single-center experience." Annali italiani di chirurgia 89: 20-23.
55. Kontio, R., et al. (2012). "Jaw bone reconstruction using CAD and stem cell technology." European Cells and Materials 24(SUPPL. 1): 33.
56. Pena Gonzalez, I., et al. (2016). "Regeneration of mandibular defects using adipose tissue mesenchymal stromal cells in combination with human serum-derived scaffolds." Journal of Cranio-Maxillofacial Surgery 44(9): 1356-1365.

Chief characteristics of included studies: Alveolar Ridge Preservation (ARP).

Study ID	Setting (Location, type of center(s), number)	Source of funding, report conflict of interest	Study design Follow-up	Operator – number/type	Population Number of patients Mean age +- SD and/or range	Risk factors	Socket location and defect definition
**D'Aquino et al. 2009**	Italy, University Hospital, Single-center	Funded by grants from the Italian MIUR (FIRB 06 n. RBIP06FH7J_006)	CCT, split mouth 12 months	Unclear / Unclear	n= 7<br>Mean age: 30.28 years<br>Range: 24-40 years<br>Sex: 6F 1M<br>10 patients lost to follow-up at 12 months and excluded of analysis	Unclear<br>No systemic diseases	Lower 3rd molar extractions<br>2- or 3 wall defects with a vertical bone loss of at least 7 mm
**Giuliani et al. 2013** (Follow-up D'Aquino et al. 2009)			36 months		n= 6		
**D'Aquino et al. 2016**	Italy, University Hospital, 8 centers	Three of the authors are components of the Medical and Scientific Division of Human Brain Wave, the company that has developed the Rigenera® protocol and Rigeneracons® disposable device.	CCT, split mouth 45-90 days	8 / Unclear	n= 35<br>Range: 25-64 years<br>Sex: 21F 14M<br>10 patients lost to follow-up at 12 months and excluded of analysis	Unclear<br>No systemic diseases	Multi-rooted tooth, extraction pre-implant therapy Interdental septum removed to create a single large alveolar bone defect.
**Kaigler et al. 2013**	USA, University, Single center	This study was funded by a Career Award for Medical Scientists from the Burroughs Wellcome Fund and funds from the NIH/NCRR. One of the authors is an employee of Aastrom Biosciences (manufacturer of the cell processing unit).	RCT, parallel, 4 groups<br>6 weeks T and C<br>12 weeks T1 and C1<br>6 months post-implant	Unclear / Unclear	n= 24<br>Range: 31-63 years<br>Sex: 13F 11M<br>1 subject (C1) lost to follow up for T_2 and T_3.	Excluded common risk factor patients at recruitment	Non-restorable tooth in need of extraction.
**Pelegrine et al. 2010**	Brazil, University Hospital, Single Center	IMPLAC and KIM Laboratories acknowledged for their support. Otherwise, none reported.	RCT, parallel, 2 groups 6 months	Unclear / Unclear	n= 13 subjects<br>T (7) C (6)<br>Mean age: 47.5±10.3 years<br>Range: 28-70 years<br>Sex: 6F 7M	Excluded common risk factor patients at recruitment	Anterior maxillary teeth (incisors or canines)- 2 teeth per patient. Sockets with severe bone loss (?) were excluded.

Y, yes; N, no; ?, unclear; RCT, randomised clinical trial; CCT, controlled clinical trial; M, male; F, female; GBR, guided bone regeneration; COLL, collagen sponge scaffold; TCR, tissue repair cells; T, test group 1; T2, test group 2; C, control group; C2, control group 2;. CAL, clinical attachment level; PD,probing depth; BV, bone volume; TV, tissue/total Volume; BS, bone surface; MSV, marrow star space volume; BVF, Bone Volume Fraction; BMD, Bone Mineral Density; BA, Bone Area; TA, Tissue Area; ARP, alveolar ridge preservation.

Chief characteristics of included studies: Alveolar Ridge Preservation (ARP). Continuation.						
**Study ID**	**Test vs Control**<br>**Source / Expansion / Time from harvesting to application**<br>**Pre- and Post-op regime**	**Atraumatic extraction**	**Flap raised**	**Primary closure**	**Healing time**	**Method of assessment of outcome**
**D'Aquino et al. 2009**	**T:** Dental Pulp Stem/progenitor cells obtained from extracted maxillary 3rd molar endorsed onto COLL (Gingistat)<br>**C:** COLL (Gingistat)<br>*Dental pulp / Yes / Delayed - 21 days*<br>Pre-op/Post-op: Professional oral hygiene one week before surgery. OHI: 0.2% Chlorhexidine (CHX) after tooth brushing, twice daily until surgery was performed.	N	Y	Y	T_1= 7 days<br>T_2= 1 month<br>T_3= 2 months<br>T_4= 3 months<br>T_5= 12 months	Clinical: CAL and PD (method not reported)<br>Radiographic: Orthopantomography (4/year)<br>Bone biopsies:<br>Histology, Immunofluorescence<br>Healing (Oedema (OED), presence of inflammation (INF)) and functionality
**Giuliani et al. 2013 (Follow-up D'Aquino et al. 2009)**					36 months	Bone biopsies:<br>Histology and Histomorphometry (n=6)<br>Synchroton X-ray Holotomography (n=1)<br>Presence of viral infections.
**D'Aquino et al. 2016**	**T:** Periosteum-derived micro-grafts obtained from gingival connective tissue+periosteum samples onto COLL (Gingistat)<br>**C:** COLL (Gingistat)<br>*Periosteum / No / Immediate - minutes*<br>Pre-op/Post-op: If abscess was present, systemic antibiotic (ot specified) given 2 weeks before the extraction. Professional oral hygiene one week before surgery. OHI: 0.2% Chlorhexidine (CHX) after tooth brushing, twice daily until surgery was performed. No antibiotics.	?	Y	N	45 / 60 /90 / 120 days after extraction<br>T_2= Implant placement.	Direct clinical measurements using a probe and a resin stent after extraction and at the time of implant placement. Horizontal and vertical ridge dimensions at 3 points.<br>Oedema (OED), presence of inflammation (INF) and Functionality.<br>Bone biopsy central part of socket, 3 mm diameter x minimum 6 mm long.<br>Histomorphometry.
**Kaigler et al. 2013**	**T:** Tissue repair cells (TRC) isolated from bone marrow aspirate (Ixymyelocel-t) endorsed onto absorbable gelatin sponge (Gelfoam®, Pfizer, New York, NY, USA) combined with a bioabsorbable collagen barrier membrane (Biomend®, Zimmer Dental, Carlsbad, CA, USA).<br>**C:** GBR (Gelfoam® soaked in 1ml sterile saline and Biomend®)<br>*Bone-marrow aspirate from posterior Ilium / Yes / Delayed: At least 12 days*<br>Pre-op/Post-op: Not reported	?	?	Y	T_1= Implant placement.<br>6 weeks (T and C) or 12 weeks (T2 and C2) after extraction<br>T_2= 3 months after T_1.<br>T_3= 3 months after T_1.	At T_1: Radiographic bone height using Emago® software,<br>Bone biopsy, μCT (TV, BV, BMD, 3d BV/TV) and histomorphometry (BMD, BVF, BA/TA).<br>Feasibility of implant placement at T_1.<br>Need for additional bone grafting at T_1, % linear implant exposure and amount of bone graft needed.<br>At T_2 and T_3: Implant survival and function<br>Crestal bone height<br>Complications
**Pelegrini et al. 2010**	**T:** Autogenous Bone Marrow graft<br>**C:** Undisturbed healing.<br>*Bone marrow graft from iliac crest punch / No / Immediate - hours*<br>Pre-op/Post-op: Not reported	?	Y	Y	T_1= 10 days<br>T_2=Implant placement. 6 months after ARP.	Titanium screw placed at time of extraction to be used as reference for measurements. Five Linear measurements of horizontal and vertical ridge dimensions using a periodontal probe. Immediately after extraction before grafting and at the time of implant placement.<br>Bone biopsy, 2 x 7 mm: for histological and histomorphometry (BMD, BVF, BA/TA) evaluation.

Chief characteristics of included studies: Ridge Augmentation (GBR).							
**Study ID**	**Setting (Location, type of center(s), number)**	**Source of funding, report conflict of interest**	**Study design Follow-up**	**Operator – number/type**	**Population Number of patients Mean age +- SD and/or range**	**Risk factors**	**Type of edentulism, Defect definition, Location**
**Correa et al. 2017^**	Brazil, Hospital, Single Center	None reported	RCT, parallel, 6 months	Unclear/Unclear	n= 10 subjects<br>Age Range: 36-52 years<br>Sex: 8F 2M	Excluded common risk factor patients at recruitment	PE, Anterior maxilla, lateral and central incisors missing with remaining bone thickness between 2 and 4 mm.<br>**Horizontal ridge augmentation.**
**Da Costa et al. 2011*^**	Brazil, University, Single center	Authors acknowledge Kopp Dental Industry Products for their support and have no with any of the manufacturers listed in this article	RCT, parallel, 6 months	Unclear/Unclear	n= 10 subjects<br>Range: 40-55 years<br>Sex: 8F 2M	Excluded common risk factor patients at recruitment	PE, Anterior maxilla.<br>**Horizontal ridge augmentation.**
**Pelegrine et al. 2016*^**	Brazil, University, Single Center	None reported	RCT, parallel, 8 months	Unclear/Unclear	n= 8 subjects<br>Mean age: 52.4.5$\pm$2.2 years<br>Range: 28-70 years<br>Sex: 6F 7M	Excluded common risk factor patients at recruitment	PE, Anterior maxilla, lateral and central incisors missing with remaining bone thickness less than 3 mm.<br>**Horizontal ridge augmentation.**

Y, yes; N, no; ?, unclear; RCT, randomised clinical trial; CCT, controlled clinical trial; M, male; F, female; FFBA, fresh-frozen bone allograft; FFBP, fresh-frozen bone particulate; PBX, particulate bone xenograft; BMAC, bone marrow aspirate concentrate; PE, partially edentulous; T, test group; C, control group; Imp, Implant placement; CBCT, Cone beam computed tomography; MT, mineralised Tissue; NMT, non mineralised tissue. *Selected for meta-analysis of clinical outcomes; ^Selected for meta-analysis of histological outcomes

Chief characteristics of included studies: Ridge Augmentation (GBR). Continuation.						
**Study ID**	**Technique**<br>**Test vs Control**<br>**Source / Expansion / Time from harvesting to application**<br>**Pre- and Post-op regime**	**Graft / (Source)**	**Membrane**	**Healing time**	**Implant / Follow-up**	**Method of assessment of outcome**
**Correa et al. 2017^**	T: BMAC + FFBA Block & FFBP<br>C: FFBA Block & FFBP<br>*Bone marrow graft from iliac crest punch / No / Immediate*<br>Pre-op/Post-op: Not reported	FFBA Block (knee) & fresh-frozen bone particulate (tibia)	N	T_1= 7 days<br>T_2= 6 months	N	Radiographic measurements:<br>CBCT volumetric changes between 7 days and 6 moths post-op<br>Average bone density<br>Bone biopsy<br>Histomorphometry (MT, NMT, MT/NMT)
**Da Costa et al. 2011*^**	T: BMAC + FFBA<br>C: FFBA<br>*Bone marrow graft from iliac crest punch / No / Immediate*<br>Pre-op/Post-op: Not reported	FFBA Block	N	T_1= Pre-op<br>T_2= 6 months post-op	Y<br>(40 implants placed).<br>No follow-up.<br>No details regarding implant system.	CBCT: Alveolar ridge width, linear measurements<br>Bone biopsy 2 x 7 mm. MT (%)
**Pelegrine et al. 2016*^**	T: BMAC + PBX (BioGen, Bioteck, Italy)<br>C: PBX (BioGen, Bioteck, Italy)<br>Bone marrow graft from iliac crest punch / No / Immediate<br>Pre-op/Post-op: Not reported	PBX: BioGen Granules 500-1000μm / Xenograft	Y<br>Equine resorbable	T_1= Pre-op<br>T_2= 4 months - Imp<br>T_3= 8 months	Y<br>4 months follow-up.<br>No details regarding implant system.	CBCT: Alveolar ridge width, linear measurements<br>Bone biopsy 2 mm in diameter:<br>Histomorphometry

Y, yes; N, no; ?, unclear; RCT, randomised clinical trial; CCT, controlled clinical trial; M, male; F, female; FFBA, fresh-frozen bone allograft; FFBP, fresh-frozen bone particulate; PBX, particulate bone xenograft; BMAC, bone marrow aspirate concentrate; PE, partially edentulous; T, test group; C, control group; Imp, Implant placement; CBCT, Cone beam computed tomography; MT, mineralised Tissue; NMT, non mineralised tissue. *Selected for meta-analysis of clinical outcomes; ^Selected for meta-analysis of histological outcomes

Chief characteristics of included studies: Sinus Augmentation (SINUS).

Study ID	Setting (Location, type of center(s), number)	Source of funding, report conflict of interest	Study design Follow-up	Operator number/type	Population Number of patients Mean age +- SD and/or range	Risk factors	Type of edentulism / Defect definition
**Nagata et al. 2012**	Japan, University, Single Center	Funding source: The Japan Society for the Promotion of Science (Project Nos. 20592370 and 23592985). Authors declared no conflict of interest.	CCT, parallel, 12 months	Unclear / Unclear	N=40 (C 15 T 25) n=18 sinus Not reported for the sample as a whole	Unclear	PE, different definitions - unclear
**Ogawa et al. 2016**	Japan, University, Single Center	Funded by the Japan Society for the Promotion of Science. Authors declared no conflict of interest	CCT, parallel, 12 months	Unclear / Unclear	Data from "some" subjects from Nagata el al. 2012 also reported here. n=39 T=23 C=16 Mean age: 58.7±3.2 years Sex: 21F 18M	Unclear	PE, advanced atrophy of the maxillary alveolar ridge and sinus floor, combined with lateral augmentation in some case
**Prins et al. 2016**	Netherlands, University, Single Center	Supported by ZonMW, the Netherlands organization for health research and development (project number 116001009). Authors declared no conflicts of interest	CCT, split mouth, 9 months post-sinus augm 3 months post implant placement	Unclear / Unclear	n= 10 subjects (C, only split mouth subjects n=6) Mean age, (range): 56 ± 7 (46-69) years Sex: 6F 4M	Excluded common risk factor patients at recruitment	Unclear, Alveolar bone height from 4mmto 8mm at the lateral maxilla. Lateral window approach.
**Ceccarelli et al 2017**	Italy, University, Single Center	Funded by 'NATO RAWINTS' (#G984961). Authors declared no conflicts of interest.	RCT, parallel, 6 months	Unclear / Unclear	n= 9 subjects (10 sinus) Mean age: 52±10 years	Unclear	PE, Unilateral or bilateral maxillary sinus floor augmentation Lateral window technique
**Kaigler et al. 2015***	USA, University, Single center	Funding was provided by ITI Foundation, the Burroughs Wellcome Fund (CAMS; DK), National Institutes of Health and Straumann Inc. Authors declared no conflicts of interest	RCT, parallel, 12 months post-implant placement	Unclear / Unclear	n= 30 subjects Mean age, (range): C: 49.1 (26-65) years T: 53 (27-66) years Sex: 20F 10M 4 subjects lost before intervention, 2 subjects lost to follow-up	Unclear	PE, Severe bone atrophy of the upper jaw. 2 stage surgery, lateral window approach. Bone height deficiencies ranged from 40% to 80%. Severe bone defects were classified as those where bone height deficiencies of >50% were present
**Sauerbier et al. 2011***	Germany, University, Single center	Supported by the Camlog Foundation, Basel, Switzerland. Technical support was given by Geistlich Biomaterials, and Harvest Technologies.	RCT, parallel, split mouth, crossover 3-4 months	Unclear / Unclear	n= 26 subjects T= 15 T2=10 C=1 45 sinus-7unilateral T= 24 T2=20 C=1 Mean age, (range): 56.6 ±8.0 year, (38.9-67.7 years) Sex: 20F 6M 14 lost to follow-up.	Excluded common risk factor patients at recruitment	PE, Posterior maxilla with a maximum of 4mm residual alveolar height.

Y, yes; N, no; ?, unclear; RCT, randomised clinical trial; CCT, controlled clinical trial; M, male; F, female; PdSC, periosteum derived stem cells; PLGA, poly (lactic-co-glycolic) acid; HA, Hydroxiapatite; b-TCP, betatricalcium phosphate; CAPCs, cultured autogenous periosteal cells; PRP, Platelet-Rich Plasma; SVF, Stromal Vascular Fraction; FFBP, fresh-frozen bone particulate; PBX, Particulate Bone Xenograft; ATh, Autogenous Thrombin; AB, Autogenous Bone; BBM, Bovine Bone Mineral; PE, Partially Edentulous; FE, Fully Edentulous; T, test group; T2, Test Group 2; C, control group; Imp, implant placement; Imp Unc, implant uncover; CBCT, cone beam computed tomography; MT. mineralised tissue; NMT, non mineralised Tissue; BVF, bone volume fraction; BMD, bone mineral density; GV, graft volume; OV, osteoid volume. *Selected for meta-analysis of clinical outcomes.

Chief characteristics of included studies: Sinus Augmentation (SINUS). Continuation.

Study ID	Technique / Test vs Control // Source / Expansion / Time from harvesting to application / Pre- and Post-op regime	Graft / (Source)	Healing time	Implant Follow-up	Method of assessment of outcome
**Non-randomised prospective controlled clinical trials**					
**Nagata et al. 2012**	**T:** CAPCs + Autogenous bone + PRP<br>**C:** Autogenous bone + PRP<br>*Periosteum harvested from gingival connective tissue in the mandibular molar region / No / Delayed – At least 6 weeks*<br>Pre-op/Post-op: Not reported	Autogenous bone from anterior region of the mandibular ramus mixed with PRP	T_1= Pre-op<br>T_2= 3 months<br>T_3= 4 months<br>T_4= 12 months	Y at T_3	CBCT at T_2 and T_4:<br>Volumetric changes<br>Bone density (Hounsfield units)<br>Bone biopsy at T_3:<br>Histomorphometry
**Ogawa et al. 2016**	**T:** CAPCs + Autogenous bone + PRP<br>**C:** Autogenous bone + PRP<br>Periosteum harvested from gingival connective tissue in the mandibular molar region / No / Delayed – At least 6 weeks<br>Pre-op/Post-op: Not reported	Autogenous bone from anterior region of the mandibular ramus mixed with PRP	T_1= Pre-op<br>T_2= 3 months<br>T_3= 5-7 months<br>T_4= 12 months	Y at T_3	CBCT at T_2 and T_4:<br>Volumetric changes<br>Bone density (Hounsfield units)<br>Insertion torque at T_3:
**Prins et al. 2016**	**T:** SVF + b-TCP (Ceros bone®)/ b-TCP+HA (Bone Ceramic®) particulate bone<br>**C:** b-TCP (Ceros bone®)/ b-TCP+HA (Bone Ceramic®) particulate bone + Ringer's lactate solution<br>*Adipose tissue from abdominal wall obtained through syringe based lipo-aspiration / No / Immediate-(hours*<br>Pre-op/Post-op: All patients received preoperative antibiotic prophylaxis, consisting of 500 mg amoxicillin. 3 times daily and for 7 days postoperatively.	b-TCP (Ceros bone®)/<br>b-TCP+HA (Bone Ceramic®)<br>Ringer's lactate solution	T_1= Pre-op<br>T_2= 5 months<br>T_3= 6 months – Imp	Y at T_3<br>3 months post Imp	OPG linear measurements<br>Bone biopsies: Histology and Histomorphometry, microCT.<br>Implant
**Randomised Controlled Trials**					
**Ceccarelli et al 2017**	**T:** PdSC + PLGA scaffold (PLGA-Fisiograft)<br>**T_2:** PdSC + PLGA/HA scaffold (PLGA/HA los)<br>*Periosteum from palatal gingival connective tissue sample / No / Immediate - mins*<br>Pre-op/Post-op: Not reported	PLGA scaffold (PLGA-Fisiograft) / PLGA/HA scaffold	T_1: 6 months<br>If 1 stage:<br>T_1: Imp Unc<br>If 2 stage:<br>T_1: Imp	Y<br>T_1: 7 implants<br>T_2: 8 implants<br>Follow-up (?)	Radiographic vertical linear measurements
**Kaigler et al. 2015***	**T:** Tissue repair cells isolated from bone marrow aspirate (Ixymyelocel-t) + b-TCP scaffold<br>**C:** b-TCP scaffold<br>*Bone marrow aspiration from iliac crest / Yes / Delayed - At least 12 days*<br>Pre-op/Post-op: Not reported	b-TCP scaffold<br>(Cerasorb, Curasan AG, Germany)	T_1= 4 months – imp plac<br>T_2= 12 moths after Imp	Y 4 months post-surgery – Straumann<br>12 months	microCT: BVF and BMD<br>CBCT linear/volumetric<br>Bone biopsy-Histomorphometry<br>Wound Healing Index.<br>Safety
**Sauerbier et al. 2011***	**T:** /parallel arm/ BMAC + BBM+ATh (n=15, 24 sinus)<br>**T2**: /split-mouth, crossover arm/ (n=10, 20 sinus)<br>T2 Group A: BMAC + BBM<br>T2 Group b: 70% BBM (70%) + AB (30%)<br>**C:** BBM (70%) + AB (30%) (n=1, 1 sinus)<br>Bone marrow graft from pelvic bone puncture / No / Immediate - hours<br>Pre-op/Post-op: Not reported	Bovine bone mineral<br>(Bio-Oss® 0.25–1mm; Geistlich Pharma AG)<br>C: Bovine bone mineral (70%) and Autogenous Bone (30%)	T_1= Pre-op<br>T_2= 3 – 4 months	Y<br>Placement 3-4 months post-surgery<br>No follow-up	Bone biopsies – Histomorphometry<br>Volume augmented bone and bone height

Y, yes; N, no; ?, unclear; RCT, randomised clinical trial; CCT, controlled clinical trial; M, male; F, female; PdSC, periosteum derived stem cells; PLGA, poly (lactic-co-glycolic) acid; HA, Hydroxyapatite; b-TCP, beta tricalcium phosphate; CAPCs, cultured autogenous periosteal cells; PRP, Platelet-Rich Plasma; SVF, Stromal Vascular Fraction; FFBP, fresh-frozen bone particulate; PBX, Particulate Bone Xenograft; ATh, Autogenous Thrombin; AB, Autogenous Bone; BBM, Bovine Bone Mineral; PE, Partially Edentulous; FE, Fully Edentulous; T, test group; T2, Test Group 2; C, control group; Imp, implant placement; Imp Unc, implant uncover; CBCT, cone beam computed tomography; MT. mineralised tissue; NMT, non mineralised Tissue; BVF, bone volume fraction; BMD, bone mineral density; GV, graft volume; OV, osteoid volume. *Selected for meta-analysis of clinical outcomes.

Chief characteristics of included studies: Periodontal Regeneration (PERIO).							
**Study ID**	**Setting (Location, type of center(s), number)**	**Source of funding, report conflict of interest**	**Study design Follow-up**	**Operator – number/type**	**Population Number of patients Mean age +- SD and/or range**	**Risk factors**	**Defect definition**
**Chen et al. 2016 Chen et al. 2018 (correction)**	China,University, Single center	Translational research grant from the Fourth Military Medical University School of Stomatology. Authors declared no conflicts of interest.	RCT, parallel, triple blind, 12 months	All patients seen by 5 operators. "Investigator 5 performed all the surgery", unclear if investigator 5 was the same operator for all patients.	n= 41 subjects Mean age, (range): T 26.05 ± 4.44 years C 30.04 ± 7.90 years Sex: 33F 8M	Excluded common risk factor patients at recruitment	Two- or Three-walled vertical intrabony defect >3 mm deep from the top of the remaining alveolar bone from radiography and clinical periodontal parameters
**Ferraroti et* al. 2018**	Italy, University, Single Center	Authors declared no conflicts of interest	RCT, parallel, double blind, 12 months	2 operators/ Periodontists	n= 29 subjects Mean age, (range): T 51.9 ± 8.4 years C 49.4 ± 9.3 years Sex: 15F 14M	Excluded common risk factor patients at recruitment	A vertical defect with residual PD ≥ 6 mm and a radiographic intrabony component ≥3 mm
**Yamamiya* et al. 2008**	Japan, University, Single Center	Grant Scientific Research from the Japan Society for the promotion of Science. Authors declared no conflicts of interest	RCT, parallel, 12 months	2 operators/ Periodontists	n= 30 subjects Mean age, (range): 55.8 ± 9.1 years Sex: 28F 2M	Excluded common risk factor patients at recruitment	Intrabony defect with PD ≥6 mm, CAL >6 mm, and an osseous defect depth estimated to be ≥3 mm when measured radiographically; ≥2 mm of keratinised gingiva (Facial)

RCT, randomised clinical trial; M, male; F, female; PDL, periodontal ligament; HA, Hydroxiapatite; PRP, Platelet-Rich Plasma; BBM, Bovine Bone Mineral; T, test group; C, control group; CAL, clinical attachment level; PD, probing depth; REC, Recession. *Selected for meta-analysis of clinical outcomes.

Chief characteristics of included studies: Periodontal Regeneration (PERIO). Continuation.				
**Study ID**	**Technique / Test vs Control**<br>***Source / Expansion / Time from harvesting to application***	**Graft / Carrier (Source)**	**Pre-op and Post-operative regime**	**Method of assessment of outcome**
**Chen et al. 2016**<br>**Chen et al. 2018 (correction)**	Type of flap not described, no membrane<br>T: PDL Cell sheets + BBM (BioOss®)<br>C: BBM (BioOss®)<br>*Periodontal ligament obtained from extracted third molars / Yes / Delayed – 4 to 5 weeks*	BBM (BioOss®)<br>Xenograft	Oral hygiene instructions, tooth cleaning and basic dental therapies if needed, such as cavity filling and occlusal adjustment at least 4-5 weeks pre-op.	Measurements at 6 sites (mesiobuccal, buccal, distobuccal, mesiolingual, lingual, and distolingual) using a stent and the same periodontal probe (type of probe not reported):<br>Clinical attachment level, Probing depth, Recession (mm)<br>Standardised radiographic assessment<br>Safety assessment
**Ferraroti et al. 2018***	Minimally Invasive Surgical Technique<br>T: Dental Pulp micrografts endorsed on a Collagen sponge<br>C: Collagen sponge endorsed with physiological solution<br>*Dental pulp obtained from extracted teeth / No / Immediate: minutes*	Collagen sponge (Condress®)	Antibiotics (875 mg amoxicillin + 125 mg clavulanic acid, 1 g b.i.d for 6 days) Analgesics (ibuprofen 600 mg, if needed). No toothbrushing/flossing for 2 weeks; 0.12% CHX rinse3/daily for 4 weeks. After 2 weeks, soft toothbrush. After 4 weeks, medium toothbrush and interdental cleaning. Weekly recalls for first month, 3-monthly thereafter.	Measurements at 6 sites per tooth using a periodontal probe (type of probe not reported):<br>Clinical attachment level, Probing depth, Recession, Intra-surgical defect morphology (bone crest to bottom of the defect (mm), and extent in mmof2- and 3- wall subcomponents)<br>Standardized radiographic assessment<br>Safety assessment
**Yamamiya et al. 2008***	T: PdSCs endorsed on HA particles (Apaceram GS3®) + PRP<br>C: HA particles (Apaceram GS3®) + PRP<br>*Periosteum samples obtained from mandibular gingival connective tissue samples / Yes / Delayed- 6 weeks*	HA granules (0.25-1 mm, Apaceram GS3®, Pentax, Japan) + PRP	Pre-op: Initial periodontal therapy: Oral hygiene instructions (FMPS<10%), SRP and occlusal adjustment (if needed).<br>Post-op: Antibiotics, Cefaclor 750 mg/day 5 days. No plaque control for 10 days, resumed after. Supragingival scaling weekly for 6 weeks then monthly recalls.	Measurements at different sites per tooth using a stent and a periodontal probe (CP-12, Hu Friedy, US):<br>CAL, PD, REC, Intra-surgical defect morphology (bone crest to bottom of the defect (mm)<br>Standardised radiographic assessment<br>Safety assessment

RCT, randomised clinical trial; M, male; F, female; PDL, periodontal ligament; HA, Hydroxiapatite; PRP, Platelet-Rich Plasma; BBM, Bovine Bone Mineral; T, test group; C, control group; CAL, clinical attachment level; PD, probing depth; REC, Recession. *Selected for meta-analysis of clinical outcomes.

Risk of bias assessment: Non randomised controlled trials

**Risk of bias assessment – Non-randomised controlled clinical trials**																																																	
ROBINS-I																																																	
Domains	Bias due to cofounding										Bias in selection of participants into the study							Bias in classification of interventions					Bias due to deviations from intended interventions								Bias due to missing data							Bias in measurements of outcome						Bias in selection of reported outcome					Overall Bias
Study ID	1.1	1.2	1.3	1.4	1.5	1.6	1.7	1.8	OR	D	2.1	2.2	2.3	2.4	2.5	OR	D	3.1	3.2	3.3	OR	D	4.1	4.2	4.3	4.4	4.5	4.6	OR	D	5.1	5.2	5.3	5.4	5.5	OR	D	6.1	6.2	6.3	6.4	OR	D	7.1	7.2	7.3	OR	D	OR & D
**Alveolar Ridge Preservation (ARP)**																																																	
D'Aquino et al. 2009	Y	N		N	N	N	N	N 16	H	U	N			N	N	H	U	Y	NI	Y	H	E	N		Y	Y	Y	NI	L		N	Y	Y	Y	N	H	E	Y	PY	PN	PY	H	U	PN	PN	PN	L		**H / E**
Giuliani et al. 2013 (Follow-up)	Y	N		N	N	N	N	N	H	U	N			N	N	H	U	Y	NI	Y	H	E	N		Y	Y	Y	NI	L		N	Y	Y	Y	N	H	E	Y	PY	PN	PY	H	U	PY	PY	PN	C	U	**H / E**
D'Aquino et al. 2016	Y	N		N	N	N	N	N	H	U	N			N	N	H	U	Y	NI	Y	H	E	N		Y	Y	Y	NI	L		NI	NI	NI	Y	NI	C	U	Y	PN	PN	PY	H	U	PY	PY	PN	C	U	**H / E**
**Sinus augmentation**																																																	
Nagata et al. 2012	Y	N		N	N	N	N	N	H	E	Y	PY	PY	N	N	H	E	N	PN	Y	H	E	N		N	N	N	N	H	E	N	NI	NI			H	E	Y	PY	PN	PY	H	U	PY	PY	N	H	E	**H / E**
Ogawa et al. 2016	Y	N		N	N	N	N	N	H	E	Y	PY	PY	N	N	H	E	N	PN	Y	H	E	N		N	N	N	N	H	E	N	NI	NI			H	E	Y	PY	PN	PY	H	U	PY	PY	N	H	E	**H / E**
Prins et al. 2016	N								L	N				N	N	C	E	Y	Y	N	L		N		Y	Y	Y		L		Y	N	N			L		NI	PY	Y	Y	C	U	N	N	N	L		C

Y, Yes; PY, Possibly yes; N, No information; PN, Possibly No; Numbers (eg 1.1) refer to question within ROBINS-I; OR, Overall Risk: L,Low / H, High / C, Some concerns;
D, Direction: E, favours Experimental / C, favours Comparator / U, Unpredictable.

Risk of bias assessment: Randomised controlled trials

Risk of bias assessment – Randomised controlled clinical trials

RoS 2.0

Domains	Randomisation					Deviations from intervention Assignment of intervention									Deviations from intervention Adhering to intervention								Missing outcome data							Outcome measurement							Bias in selection of reported outcome					Overall Bias	
Study ID	1.1	1.2	1.3	OR	D	2.1	2.2	2.3	2.4	2.5	2.6	2.7	OR	D	2.1	2.2	2.3	2.4	2.5	2.6	OR	D	3.1	3.2	3.3	3.4	3.5	OR	D	4.1	4.2	4.3	4.4	4.5	OR	D	5.1	5.2	5.3	OR	D	Overall Risk	Direction
**Alveolar Ridge Preservation (ARP)**																																											
Kaigler et al. 2013	Y	Y	N	L	U	Y	Y	N			NI	PN	C	U	Y	Y	Y	Y	Y	Y	C	U	Y					L		N	N	N			L	U	PY	N	N	L	U	C	U
Pelegrine et al. 2010	NI	NI	Y	H	U	PY	PY	N			NI	PN	H	U	Y	Y	Y	Y	Y	Y	C	U	Y					L		N	N	PY	PY	NI	C	U	NI	PN	PN	C	U	H	U
**Ridge Augmentation**																																											
Correa et al. 2017	Y	NI	NI	C	U	Y	PY	N			NI	PN	H	U	Y	PY	Y	Y	Y	N	C	U	Y					L		PN	N	PY	PY	PN	C	U	NI	PY	PY	H	E	H	U
Da Costa et al. 2011	NI	NI	Y	H	U	Y	PY	N			NI	PN	H	U	Y	PY	Y	Y	Y	N	C	U	Y					L		PN	PY	PY	PY	NI	H	E	NI	PY	PY	H	E	H	E
Pelegrine et al. 2016	Y	NI	NI	C	U	Y	PY	N			NI	PN	H	U	Y	PY	Y	Y	Y	N	C	U	Y					L		PN	N	PY	PY	PN	C	U	NI	PY	PY	H	E	H	U
**Sinus augmentation**																																											
Ceccarelli et al. 2017	Y	NI	NI	C	U	N	PY	PN	N	PY	NI	N	H	U	NI	PY	Y	PN	Y	N	H	U	Y					L		NI	NI	PN	PY	NI	H	U	NI	NI	NI	H	U	H	U
Kaigler et al. 2015	Y	NI	PY	C	E	Y	PY	N			NI	PN	C	U	Y	NI	PY	Y	Y	N	C	E	Y					L		N	N	NI	PY	NI	L		Y	N	N	L		C	U
Sauerbier et al. 2011	Y	PY	N	L		Y	PY	N			NI	PN	C	U	Y	NI	PY	Y	Y	N	C	E	N	N	NI	NI	N	H	U	PY	PY				H	E	NI	PY	PY	H	U	H	U
**Periodontal Regeneration**																																											
Chen et al. 2016	Y	Y	N	L		N	N				Y		L		N	N		N	Y		L		Y					L		N	N				L		Y	N	N	L		L	
Ferraroti et al. 2018	Y	Y	N	L		N	N				Y		L		N	N		N	Y		L		Y					L		N	N				L		Y	N	N	L		L	
Yamamiya et al. 2008	Y	NI	N	C		Y	Y	N			NI	N	C		Y	Y	Y	PN	Y		C		Y					L		N	N				L		Y	N	N	L		C	

Y, Yes; PY, Possibly yes; N, No information; PN, Possibly No; Numbers (eg 1.1) refer to question withir RoS 2.0; OR, Overall Risk: L,Low / H, High / C, Some concerns;
D, Direction: E, favours Experimental / C, favours Comparator / U, Unpredictable

Alveolar Ridge preservation: Outcome synthesis.

Study ID	Comparison	Changes in alveolar ridge dimensions	Histology	Changes in keratinized tissue dimensions and soft tissue volume	Other outcomes<br>Complications
**D'Aquino et al. 2009**	T: SC + COLL<br>C: COLL	**CAL gain – Vertical** (does not report method of measurement)<br>T: 6.2±2.3 mm<br>C: 4.4±1.2 mm<br>**% regeneration radiographic** (method not reported- OPG as example since to take into account both horizontal and vertical dimensions) at 12 months:<br>**C (n):** **T(n):**<br>Code 0= 1 Code 0= 0<br>Code 1= 3 Code 1= 3<br>Code 2= 3 Code 2= 3<br>Code3= 0 Code 3= 2<br>**Difference T v C**<br>No change = 1, 1 code up for T = 5, 2 codes up for T= 1<br>P<0.01 at subject level for all subjects.<br><br>Code 0 No regeneration<br>Code 1 30% regeneration<br>Code 2 70% regeneration<br>Code 3 Complete regeneration.	T site samples were made up of well-organized and well vascularized bone with a lamellar architecture surrounding the Haversian channels.<br>At C sites bone was immature, with fibrous bone entrapped among new lamellae, incomplete and large Haversian channels and evidence of bone reabsorption. In all cases the collagen sponge was completely reabsorbed.	n/a	Cells strongly positive to CD34 and F1k-1<br>Report cell yield as sufficient (no measurement reported)<br>No difference in OED or INF at 7 days: 0% complications/infection. No pain – lack of need for analgesic medication. No bleeding, no swelling or other side effects were observed. Quality of life, chewing, oral cavity and relative functions remained optimal in all the cases up to 12 months.<br>**Radiographic outcomes:**<br>T1= 2 months: higher rate of mineralization at Test group<br>T2= 2 months: cortical margin reached the<br>(CEJ) level of the second molar at test group but not at control group in any subject – no measurements reported<br>T3= 3 months: sites were completely regenerated<br>and that the cortical level was much higher than at the C sites. (no p value or quantitative measurement provided).<br>IF: Significant differences were observed for BMP-2 and VEGF which were expressed at much higher levels<br>(p<0.001) in the T samples (no p value or quantitative measurement provided).
**Giuliani et al. 2013 (Follow-up)**	T: SC + COLL<br>C: COLL	**PD – Vertical** (does not report method of measurement)<br>T: 6.3+-2.1 mm<br>C: 4.5+-1.2 mm. p < .001	**Histomorphometric analysis**<br>BV (μm^3)<br>T 1.10±0.3 ($x10^8$)<br>C 0.53 +- 0.31 ($x10^8$)<br>*p* <.001<br>BS/BV (mm^{-1})<br>T 15±1 C 32±0 *p* <.001<br>BS/TV (%)<br>T 79.8±10.3 C 47.**6**±**7**.6 *p* <.01<br>MSV (%)<br>T 11.2 ±2.3 C 52.3±1.5 *p*=.001	n/a	No morbidity/complications. Normal clinical appearance. Adequate function. No viral infections.<br>**Radiographic outcomes:**<br>Three years after surgery, T sites were completely regenerated and had better vertical bone height with respect to the C sites (no p value or quantitative measurement provided).<br>***3D holotomography-based morphometric analysis***<br>BV ($\mu m3$) BS/BV (mm^{-1}) (%)<br>T 1.10 ±0.18 ($x10^8$) T 18 ±6<br>C 0.49 ±0.32 ($x10^8$) *p*=.003 C 55 ±29 *p*= .004<br>BS/TV (%) Mean BTh (μm)<br>T 62.4 ±11.7 T 119±27<br>C 24.8 ±15.2 *p*= .001 C 48±29 *p*= .001<br>Mean BNr (mm^{-1}) Mean BSp (μm)<br>T 5.3 ±0.4 T 70.1 ±17.6<br>C 5.2 ±0.9 *p*= .680 C 155.0±61.2 *p*= .003<br>**Synchrotron Radiation-Based Holotomography**

					Mean percentage of high BMD T 57.7±15.0% C (69.3 ± 6.7%).
**D'Aquino et al. 2016**	T: DPSCs + COLL<br>C: COLL	**From direct clinical measurements. Vertical.**<br>T_2: Resorption was in T sites 36.5% less than C sites.<br>T= -0.82 mm (from fig 2B)<br>C= - 1.3 mm (from fig 2B)<br>P ≤ 0.001<br>**From direct clinical measurements. Horizontal**<br>T_2: Resorption in the T sites was 38.3% less than in C sites.<br>T= -1.8 mm (fig 2A)<br>C= - 3 mm (fig 2A)<br>P ≤ 0.001	n/a	n/a	No difference in OED or INF at 7 days. 0% complications/infection. 7 subjects required painkillers. No bleeding, no swelling or other side effects were observed. Quality of life, chewing, oral cavity and relative functions remained optimal in all the cases up to 12 months.<br>**Histology:**<br>Ossification process was much faster in T compares to C sites. At 45 days, C was characterized by the presence of inflammatory cells and no bone formation was present.<br><br>T exhibited bone formation already at 45 days, with an increase of calcified matrix at 60, 90 and 120 days with respect to Control group where the organic matrix is more evident.
**Kaigler et al. 2013**	T: BMSC + GEL + MEM<br>C: SALINE + GEL + MEM	**Linear radiographic bone height**<br>(percentage of the regenerated bone height over the height of the initial defect)<br>Mean(%) C 55.3 T 78.9<br>C1 74.6±3.3 T1 80.1±2.0<br>Mean diff CI<br>C – T 23.6 (6.02,41.09) *p*=0.01<br>C1 – T1 5.4 (-12.11, 22.95) *p*=0.28	**BVF (%)**<br>Mean C 13±3 T 28±8<br>C1 24 T1 30<br>Mean diff (CI)<br>C-T 15 (−3, 34) p=0.07<br>C1-T1 5 (−14, 24) p=0.30<br>**BMD (mg/cc)** Mean<br>C 85.5 ±46.3<br>T 195.0±63.3<br>C1 146.6 T1 186.8<br>Mean diff (CI)<br>C-T 109.5 (−28.6, 247.5) p=0.08<br>C1-T1 40.2 (97.8, 178.3) p=0.29<br>**Bone area/tissue area (BA/TA) %**<br>Mean C 19.6±4.2 T 33.5±9.1<br>C1 35.1±3.2 T1 35.2±8.9<br>Mean diff (CI)<br>C-T 13.9 (−5.3, 33.2) p=0.09<br>C1-T1 0.2 (−19.1, 19.4) p=0.49	**Mean (keratinized) gingival tissue width,mm (SD)**<br>C 4.8 (2.9) T 3.8 (0.7)<br>C1 5.2 (1.2) T1 4.7 (1.8)<br>**Mean (keratinized) gingival thickness, mm (SD)**<br>C 1.8 (1.2) T 1.5 (0.5)<br>C1 1.8 (0.7) T1 1.1 (0.2)	No morbidity/complications.<br>100% implant placements.<br>100% survival at 3, 6 months and up to 12 months (in text)<br>**Mean % linear implant exposure (CI)**<br>C 29.2% (−1.2, 60) T 5.1% (−8.5, 18.7) p=0.04<br>C1 25.3% (−0.9, 51.5) T1 3.8% (−6.1, 14) p=0.03<br>**Cases Requiring secondary bone grafting**<br>C 5 T 2 C1 3 T1 2<br>**Mean amount additional graft used (cc) – (CI)**<br>C 0.23 (0.02, 0.44) T 0.09 (−0.01, 0.2) p=0.08<br>C1 0.08 (−0.02, 0.2) T1 0.05 (−0.05, 0.16) P=0.31<br>**In vitro osteogenic potential (AP activity) and mineralization (Von Kossa) ability of TRCs were correlated with the clinical outcome measures of BMD and BVF for each respective T population.** There was a positive correlation between AP and BVF (r = 0.56, p = 0.058) and a statistically significant positive correlation between AP and BMD (r = 0.58; p = 0.049). Positive correlations with in vitro mineralization ability and BMD and BVF measures were not statistically significant.
**Pelegrine et al. 2010**	T: BMAC<br>C: Undisturbed healing	**External vertical measurement**; from the head of the screw to the coronal border of the alveolar buccal plate.<br>Baseline Mean / 6 months / Difference<br>T 2.6 mm ± 0.45 / 1.98 mm ± 0.63 / 0.62 mm ± 0.51<br>C 2.67 mm ± 0.4 / 1.29 mm ± 0.4 / 1.17 mm ± 0.26<br>P value / 0.028 / 0.016<br><br>Baseline Range / 6 months / Difference<br>T 2-3 mm / 1-2.5 mm / 0-1.67 mm	**% of Mineralised bone**<br>Mean ± SD (range)<br>T 45.47% ± 7.21. (39, 56.3)<br>C 42.87% ± 11.33 (37, 50.5)<br>P+0.36		No morbidity/complications.<br>No or minimal discomfort with regard to the bone marrow Harvesting (no quantitative/validated measure).<br><br>**Cases Requiring secondary bone grafting**<br>C 5 T 0

		C 2-3 mm 1-2 mm 1-1.5 mm<br><br>***Internal vertical measurement***: from the most apical end of the socket to the coronal border of the buccal plate<br>Baseline Mean 6 months Difference<br>T 10.26mm ±1.5 0.2mm ± 0.22 10.06 mm ± 1.1<br>C 10.71mm ±0.81 0.27mm ± 0.23 10.44 mm ± 0.84<br>P value= 0.33 0.72<br>Baseline Range 6 months Difference<br>T 9-12 mm 0-0.5 mm 9 - 11.83 mm<br>C 9.5-12 mm 0-0,.5 mm 9.5-12 mm<br>**Clinical horizontal measurement** (CHM): distance between the buccal and the lingual borders of the alveolar plates<br>Baseline Mean 6 months Difference<br>T 7.38 mm ± 0.7 6.24 mm ± 0.58 1.14 mm ± 0.87<br>C 7.38 mm ±0.49 4.92 mm ± 0.86 2.46 mm ± 0.4<br>P value= 0.008 0.014<br>Baseline Range 6 months Difference<br>T 6.5 - 8.5 mm 5.5-7 mm 0.5 - 3 mm<br>C 7 – 8.25 mm 4-6,.5 mm 1.75 - 3 mm<br><br>**Alveolar Thickness loss (%)**<br>Mean±SD (range)<br>T 13.61± 12.5 (7.14, 35.42) C 31.35% ±11.88 (20.24, 42.86)<br>P=0.006<br><br>At time of implant placement only:<br>**Vestibular thickness loss** (VTL): the distance from the head of the screw to the buccal plate. (mm)<br>T 0.9± 0.81 (0.5, 2) C 1.83 ± 0.77 (1.5, 2.75) P=0.01<br>**Palatal thickness loss**: CHM minus VTL.<br>T 0.17± 0.36 (0, 1) C 0.5 ± 0.53 (0.25, 1) P=0.018			

GBR, guided bone regeneration; COLL, collagen sponge scaffold; SC, Stem/progenitor cells; GEL, absorbable gelatin sponge; MEM, bioabsorbable collagen barrier membrane;

T, test group 1; T2, test group 2; C, control group; C2, Control Group 2;. CAL, Clinical Attachment Level; PD, Probing depth; BV, Bone volume; TV, Tissue/Total Volume; BS, Bone surface; MSV, Marrow star Space Volume; BVF, Bone Volume Fraction; BMD, Bone Mineral Density; BA, Bone Area; TA, Tissue Area.

Lateral Ridge Augmentation: Outcome synthesis.

Study ID	Comparison	Changes in vertical alveolar ridge dimensions- mid buccal vs proximal if reported<br>Changes in horizontal alveolar ridge dimensions	Histology (%)	Changes in keratinized tissue dimensions<br>Changes in soft tissue volume	Other outcomes<br>Complications
**Correa et al. 2017**	T: BMAC + FFBA Block & FFBP<br>C: FFBA Block & FFBP	**Volume of Grafted area (mm³)**<br>Volume at T_1 Volume at T_2 P value<br>C: 612.69 ± 245.57 457.30 ± 189.65 0.098<br>T:711.17 ± 215.55 542.95 6± 141.60 0.017<br>P value 0.519 0.442	MT (%)<br>C: 37.77 ± 15.19<br>T: 43.95 ± 10.94.<br>P=0.464<br>NMT (%)<br>C: 62.15 ± 14.90<br>T: 56.30 ± 10.72<br>P=0.464	Not reported	No complications reported<br>**Mean Bone Density of the Grafted Bone Blocks**<br>(in Hounsfield Units, HU)<br>Density at T_1 Density at T_2 P value<br>C: 238.4 ± 68.70 507.8 ± 49.90 0.002<br>G: 219.3 ± 20.00 476.4 ± 81.50 0.002<br>P 0.464 0.916<br>**Mean Bone Density of the Grafted Bone Blocks**<br>(in Hounsfield Units, HU) at T2 according to Graft Region<br>Palatine Region* Buccal Region P-value<br>C: 554.50 ± 66.30 267.90 ± 61.20 0.002<br>T: 483.90 ± 115.00 420.80 ± 110.90 0.172<br>P 0.464 **0.0283**
**Da Costa et al. 2011**	T: BMAC + FFBA<br>C: FFBA	**Tomographic parameters comparison (mm)**<br>Group T1 T2 P value<br>T 4.3 8.9 0.05<br>C 4.8 6.9 0.05<br>**Alveolar thickness gain.**<br>Group (mm) (%)<br>T 4.6 ± 1.43 118.23 ± 56.93<br>C 2.15 ± 0.47 49.91 ± 20.24<br>P value 0.002 0.002	MT (%)<br>C: 41.4. ± 12.5<br>T: 60.7 ± 16.18<br>P=0.019	Not reported	No morbidity/complications reported.<br>100% implants placed.
**Pelegrine et al. 2016**	T: BMAC + PBX<br>C: PBX	**Alveolar thickness gain (mm)**<br>Group (T_2) (T_3)<br>T 3.79 ± 0.52 4.09 ± 1.33<br>C 3.78 ± 1.35 4.34 ± 1.58	T_2 onl. MT (%)<br>C: 52.30 ± 16.78<br>T: 65.04 ± 20.98<br>P=0.13<br>NMT (%)<br>C: 47.70 ± 16.78<br>T: 34.96 ± 10.38<br>P=0.18	Not reported	100% implant survival 4-month post-placement.

Sinus Augmentation: Outcome synthesis.

Study ID	Comparison	Changes in vertical alveolar ridge dimensions<br>- mid buccal vs proximal if reported<br>Changes in horizontal alveolar ridge dimensions	Histology (%)	Other outcomes<br>Complications
**Nagata et al. 2012**	T: CAPCs + AB + PRP<br>C: AB + PRP	**Volume changes were calculated as ratios of the augmented bone volumes at 1Y to 3 M after the graft. Data reported only for T (n=4) an C (n=3).**<br>T: 0.71 C=0.66 No statistical significance. Both groups reduced by approx. 35% from 3 months to 12 months.	**Alkaline Phosphatase positive area**<br>T (n=4) 24.4% C (n=4) 7.3%. p<0.05<br>**Tartrate-resistant acid phosphatase activity (TRAP)+ cells/square**<br>T (n=4) 6.5 C (n=4) 0.4%. p<0.001	No adverse events attributable to the use of CAPCs were found. One case with a background of chronic sinusitis showed progressive alveolar resorption after the sinus lift procedure. One site of partial exposure of the titanium-mesh and one site of partial fibrosis were observed on grafts.
**Ogawa et al. 2016**	T: CAPCs + AB + PRP<br>C: AB + PRP	**Volume changes were calculated as % of the augmented bone volumes at 1Y to 4M after the graft. Data reported only for T (n=25) and C (n=15).**<br>T: 73.3± 8% C=73.2± 11% No statistical significance.. Both groups reduced by approx. 30% from 4 months to 12 months.		**Insertion torque (Ncm) T n=30 n=8**<br>T: 29.7± 9.6 C= 16.3± 7.8
**Prins et al. 2016**	T: SVF + b-TCP / b-TCP+HA<br><br>C: b-TCP/bTCP+HA + Ringer's lactate solution	**Mean vertical radiographic increase – 5 months:**<br>b-TCP 10.2 ± 1.5 mm b-TCP + SVF 9.9 ± 1.3 mm<br>bTCP+HA 12.4 ±1.6 mm b-TCP+HA+SVF 12.1 ± 1.6 mm	**BV/TV** T: 15.2% ± 4.7% C: 13.3% ± 3.0%<br>**GV/TV** T: 17.7% ± 8.3% C: 24.4% ± 8.6%<br>**OV/TV** T: 0.6% ± 0.4% C: 0.4% ± 0.2%<br>**BV/TV** b-TCP + SVF 16.4% ± 5.2% b-TCP 12.0% ± 2.6%<br>b-TCP+HA+ SVF 15.1% ± 2.3% b-TCP+HA 14.7% ± 3.2%)<br>**GV/TV** b-TCP + SVF 17.4% ± 9.4% b-TCP 29.6% ± 8.2%<br>b-TCP+HA+ VF 18.5% ± 3.7% b-TCP+HA 19.1% ± 5.9%)<br>**OV/TV** b-TCP + SVF 0.8% ± 0.3% b-TCP 0/2% ± 0.1%<br>b-TCP+HA+ VF 0.9% ± 0.8% b-TCP+HA 0.5% ± 0.2%)<br>*All above at 6 months.*	**Implant survival at 3 months T:** 15/16 C 22/22.<br><br>**Percentages BV/TV (micro-CT)**<br>b-TCP + SVF 18.4% ± 6.8% b-TCP 11.2% ± 0.9%<br>b-TCP+HA+ SVF 18.0% ± 2.4% b-TCP+HA 16.2% ± 5.4%)<br>T: 19.5% ± 3.8% C: 13.7% ± 4.4%<br>**Percentages GV/TV (micro-CT)**<br>b-TCP + SVF 9.7% ± 3.4% b-TCP 10.6% ± 3.4%<br>b-TCP+HA+ SVF 12.1% ± 0.5% b-TCP+HA 11.7% ± 2.3%)<br>T: 10.5% ± 3.6% C: 14.0% ± 3.6%
**Ceccarelli et al 2017**	T: PdSC + PLGA<br>T_2:PdSC+PLGA/HA	**Mean vertical radiographic increase:**<br>T: 8.2 ± 3.5 mm T_2: 8.8 ± 3.0 mm P=0.52		
**Kaigler et al. 2015**	T: SC + b-TCP scaffold<br>C: b-TCP scaffold	**Mean baseline alveolar bone height in mm (range):**<br>C: 5.0 (2.5-6.2) T: 3.5 (2.1-6.1)<br>**Increase in bone height mean (in mm ± SD) - 4months**<br>C: 12.8 ± 2.8 T: 12.2 ± 3.3<br>**Increase in bone volume (in mm^3 ± SD) - 4 months**<br>C: 2.1 ± 0.9 T: 1.8 ± 1.0		Similar outcomes on patient-reported outcome measures and soft tissue healing post-operatively (Wound Healing Index). T: 1 graft failure due to sinusitis and 1 implant failure.<br>**Implant survival 12 months:** C (20/20) T (18/19).<br>**Bone Volume Fraction (± SD) - 4 months - (micro-CT)**<br>C: 0.43 ± 8.1 T: 0.49 ± 7.2<br>**Bone Mineral Density (± SD) - 4 months - (micro-CT)**<br>C: 0.79 ± 0.05 T: 0.78 ± 0.02
**Sauerbier et al. 2011**	T: BMAC + BBM+ATh<br>C: BBM (70%) + AB (30%)	**Volumetric radiographic gain *mL***<br>**(39 samples – T=28 C=11)**<br>T: 1.74 ± 0.69 C: 1.33 ± 0.62 p=0.02<br>*Diff* 0.41 *mL* 95% & CI (0.13-1.04 *mL*)	**New Bone Formation (± SD) – 3/4 months** p=0.333<br>C: 14.3% ± 1.8 T: 12.6% ± 1.7*diff* 1.7% (-4.6%, 1.2%)<br>**Measured fraction of biomaterial (± SD) – 3/4 months**<br>C: 19.3% ± 2.5 T: 31.3% ± 2.7 p<0.0001 *diff* 12%<br>**Marrow space (± SD) – 3/4 months**<br>C: 57.7% ± 2.3 T: 54.4% ± 2.2 *diff* 3.3% p=0.137	No occurrence of pain, hematoma, or infection at any time after bone marrow aspiration and sinus floor augmentation<br>C: BBM + AB), 1 injury of the inferior alveolar nerve, from the harvesting of the retromolar bone. 2 other infections of bone harvesting site.

Periodontal regeneration: outcome synthesis.

Study ID	Comparison	Subjects (n)		Baseline Parameters (mean ± SD mm)		CAL gain (mean ± SD mm) PD reduction (mean ± SD mm)		Bone fill (mean ± SD mm)		Other outcomes
		Cell-based therapy	Control Group	Cell-based therapy Group	Control Group	Cell-based therapy Group	Control Group	Cell-based therapy Group	Control Group	
**Chen et al. 2016 Chen et al. 2018 (correction)**	T: BBM (BioOss®) + Cell sheets<br>C: BBM (BioOss®)	20<br>18F 2M	21<br>15F 6M	CAL 5.15 ± 1.52<br>PD F 6.43 ± 1.92<br>PD L/P 6.25 ± 1.36<br>BD 7.20 ± 2.65	CAL 5.28 ± 1.60<br>PD F 5.68 ± 1.59<br>PD L/P 5.86 ± 1.43<br>BD 7.19 ± 1.87	CAL 4.42 ± 1.19*<br>***Diff* 0.73**<br>PD F 3.80 ± 1.03*<br>***Diff* 2.63**<br>PD L/P 4.20 ± 0.86*<br>***Diff* 2.25**	CAL 5.07 ± 1.48*<br>***Diff* 0.21**<br>PD F 3.88 ± 0.77*<br>***Diff* 1.80**<br>PD L/P 3.79 ± 0.55*<br>***Diff* 2.07**	BD 4.49 ± 2.03^<br>*Diff* 2.71	BD 4.80 ± 1.41^<br>*Diff* 2.39	No adverse events<br>Recession (mm± SD)<br>T Facial B 0.70 ±1.09 R*1.28 ±0.82<br>C Facial B 0.62 ±0.89 R*1.54 ±0.96<br>T L/P B 0.73 ±0.87 R*1.23 ±0.92<br>C L/P B 0.52 ±0.85 R*1.38 ±1.37
**Ferraroti et al. 2018**	T: DPSCs + COLL<br>C: COLL + SAL	15<br>7F 8M	14<br>8F 6M	CAL 10.0 ± 1.6<br>PD 8.3 ± 1.2<br>BD 6.4± 1.4	CAL 9.4 ± 1.5<br>PD 7.9 ± 1.3<br>BD 5.6± 1.0	CAL 4.5 ± 1.9^<br>PD 4.9 ± 1.4^	CAL 2.9 ± 2.2^<br>PD 3.4 ± 1.7^	3.9 ± 1.2^	1.6 ± 1.1^	Sites PPD^ <3mm T 66.7% C 14.3%<br>Sites PPD^ >6mm T 0% C 14.3%<br>CAL gain^ >4mm T 73.3% C 28.5%<br>Recession (mm)^ ± SD<br>T -0.4 ± 1.1 C -0.5 ± 0.9
**Yamamiya et al. 2008**	T: PDSCs + HA +PRP<br>C: HA + PRP	15<br>14F 1M	15<br>14F 1M	CAL 8.1 ± 1.2<br>PD 7.7 ± 1.1<br>BD 5.3± 1.1	CAL 8.0 ± 1.3<br>PD 7.6 ± 1.1<br>BD 4.9± 1.4	CAL 3.9 ± 1.6^<br>PD 4.8 ± 1.1^	CAL 2.7 ± 1.3^<br>PD 4.3 ± 1.1^	4.9 ± 1.2^	3.2 ± 1.1^	CAL gain (%)^ ± SD<br>T 83.5% ± 31.7 C 55.0% ± 21.9<br>Recession (mm± SD)^<br>T -0.9 ± 1.5 C -1.7 ± 1.3

B, Baseline; R, Reassessment; CAL, Clinical attachment level; PD, Probing Depth; F, Facial; L/P, Lingual/Palatal; R, Recession; BD, Radiographic Bone defect; DPSCs, Dental Pulp Stem Cells; COLL, Collagen sponge; SAL, Physiological solution; IQ, interquantile.

*Outcome at 3 months ^Outcome at 12 months

Full description of techniques for clinical application of cell-based therapy.

SUMMARY OF CELL-BASED TECHNIQUES

Alveolar Ridge preservation		
**Study ID**	**Cell-based technique**	**Description as published**
**D'Aquino et al. 2009**<br>**Giuliani et al. 2013 (Follow-up)**	**Cell source:** Dental pulp from extracted tooth<br>**Culturing/Expansion:** Yes<br>**Scaffold/Carrier:** Collagen sponge<br>**Time from harvesting to application:**<br>Delayed - At least 21 days	Patients were therefore subjected to the extraction of the upper (maxillary) molars and the pulps harvested. Teeth were washed in 0.2% CHX solution, the pulp chamber opened using a surgical drill and the pulp collected. Then, the pulp was rinsed in 1.5 ml saline solution and mechanically dissociated. After dissociation, cells were filtered through a 70µm strainer and cultured in α- minimal essential medium (MEM) (Cambrex, Charles City, IA, USA) with 20% FBS (Invitrogen, Italy) and the medium changed twice a week. At day 21, cells were detached and gently endorsed with a syringe onto a collagen sponge scaffold (Gingistat, Italy). The sponge-cell implant was used to fill the space left by the extraction procedure. A flap of gum was then sutured as a tendon in order to avoid any contact with the oral cavity. A suture was placed at the distal portion of the second molar and the others were placed at the interdental papillae and at the posterior end of the incision.
**D'Aquino et al. 2016**	**Cell source:** Periosteum from gingival connective tissue sample<br>**Culturing/Expansion:** No<br>**Scaffold/Carrier:** Collagen sponge<br>**Time from harvesting to application:** Immediate - minutes	To disaggregate the periosteum samples, a Rigenera® protocol was performed, based on the use of a disposable medical device called Rigeneracons® (Human Brain Wave, Italy), a biological disruptor of human connective tissues able to filter and select progenitor cells with a size of 50 micron. The device allows to obtain autologous micro-grafts ready to use in a safe and easy manner without extensive manipulation. Every device owns a grid with 100 hexagonal holes and any hole is embraced by six micro-blades designed for efficient cutting of hard and soft tissues. The researchers collected a small piece of connective tissue (1-2 mm and up to 10mm) that was inserted in the Rigeneracons® device with 1 ml of physiological solution. After this, the tissues are disaggregated after inserting the filter in the Rigenera® machine which activates rotation (75 r/min and 15 Ncm) and achieves mechanical disruption. After 2 minutes, the micro-grafts suspension are collected with a syringe. The cell suspension was then used to soak a collagen sponge for 10 minutes in order to build a bio-complex that was directly grafted on the alveolar socket.
**Kaigler et al. 2013**	**Cell source:** Bone marrow aspiration -Iliac crest<br>**Culturing/Expansion:** Yes<br>**Scaffold/Carrier:** Gelatin sponge<br>**Time from harvesting to application:**<br>Delayed - At least 12 days	Bone marrow aspiration of the posterior ilium under conscious sedation and local anesthetic. Collected marrow was transferred to a sterile blood bag and then inoculated into a bioreactor, which is a proprietary computer-controlled, automated cell processing unit (Aastrom Biosciences). The cell cassette within this system provides a functionally closed, sterile environment in which cell production occurs. The fluid pathway in the cell cassette includes the cell growth chamber, a medium supply container, a mechanism for medium delivery, a waste medium collection container, and a container for the collection of harvested cells. The culture medium consists of Iscove's modified Dulbecco's medium (IMDM), 10% fetal bovine serum, 10% horse serum, 5 µM hydrocortisone. This system incorporates single-pass perfusion in which fresh medium flows slowly over cells without retention of waste metabolites or differentiating cytokines. After cultivation for 12 days at 37°C and 5% CO2, the TRC product was harvested by trypsinization, resuspended, and collected into a sterile bag where it was stored until the time of transplantation.<br>During surgery, 1 ml of the cell suspension (approximately 1.5 × 10^7 cells/ml) was placed onto an absorbable gelatin sponge (Gelfoam®, Pfizer, New York, NY, USA) sized to a dimension of approx. 2 cm^3 and delivered in the extraction socket. A bioabsorbable collagen barrier

		membrane (Biomend®, Zimmer Dental, Carlsbad CA, USA) was placed over the sponge to contain the cell construct and the tissues were closed.
**Pelegrine et al. 2010**	**Cell source:** Bone marrow aspiration - Iliac crest<br>**Culturing/Expansion:** No<br>**Scaffold/Carrier:** Gelatin sponge<br>**Time from harvesting to application:** Immediate - hour (s)	Before the dental procedure, the patients had 5 ml of bone marrow harvested from the iliac by hematologists from the Hematology and Blood Transfusion Center of the University of Campinas – UNICAMP, SP, Brazil. To obtain the bone marrow, the hematologists generated, after local anesthesia with 2% lidocaine, a punch in the posterior upper iliac crest using a 40x12mm needle. The bone marrow was maintained in heparin (1ml) to avoid blood coagulation. The patients had their sockets grafted with an autologous bone marrow aspiration applied directly and not endorsed in any scaffold. After a periosteal releasing incision, the mucosal flap was sutured with 5-0 nylon sutures to initiate primary wound healing.
**Lateral Ridge Augmentation**		
**Correa et al. 2017**	**Cell source:** Bone marrow aspiration -Iliac crest<br>**Culturing/Expansion:** No<br>**Scaffold/Carrier:** FFBA&FFBP<br>**Time from harvesting to application:** Immediate – hour(s)	Thirty milliliters of bone marrow were aspirated from the posterior iliac bone crest and immediately inserted into a collection bag, to which 8 mL of an acid citrate dextrose anticoagulant solution was added. After homogenization, the contents were passed through a filter in the collection bag, where all the fibrous material was retained. The filtered contents were then withdrawn by syringes and transferred into the centrifugation vials. The Bone Marrow Aspirate Concentrate (BMAC) System provided by Harvest Terumo BCT (Terumo Medical do Brasil, Brazil) was used to obtain the BMAC. This system consists of an automatic centrifuge (SmartPReP 2) and a processing kit. The vials containing the filtered marrow were then centrifuged at 2400 rpm for 14 minutes, thus forming 2 evident phases within the vial: an aqueous phase (supernatant plasma) and a solid phase (concentrated sediment; Fig. 1). Immediately after plasma removal, the concentrate (4 mL) was aspirated with a sterile syringe, where it was stored until time of use.<br>The blocks were impregnated with BMAC by dripping. Fixation was performed by titanium alloy screws 1.5mmin diameter and 12.0 mm in length (Neodent, Brazil), and the surgical gaps were filled with 1 mL of fresh homologous bone particulate.
**Da Costa et al. 2011**	**Cell source:** Bone marrow aspiration -Iliac crest<br>**Culturing/Expansion:** No<br>**Scaffold/Carrier:** FFBA<br>**Time from harvesting to application:** Immediate - hour (s)	Before the bone augmentation procedure, the patients in the experimental group had 4.0 mL of bone marrow aspirated from the iliac by hematologists. For bone marrow aspiration, a punch in the posterior upper iliac crest using a 40 x 12 mm needle was performed after local anesthesia with 2% lidocaine. To avoid blood coagulation, the bone marrow aspirate was maintained in heparin (1.0 mL). The blocks, embedded with the bone marrow aspirate, were adapted to the bone defect and fixed with titanium screws.
**Pelegrine et al. 2016**	**Cell source:** Bone marrow aspiration -Iliac crest<br>**Culturing/Expansion:** No<br>**Scaffold/Carrier:** PBX<br>**Time from harvesting to application:** Immediate - hour (s)	Bone marrow was harvested and processed in the operating room using the BMAC system (Bone Marrow Procedure Pack; Harvest Technologies, USA). In an outpatient setting and using local anesthesia (2% xylocaine without a vasoconstrictor), 30 mL of bone marrow aspirate was obtained from all patients via a puncture 2 cm laterocaudally from the upper posterior iliac crest, using a bone marrow needle (included in the pack) and heparinized 30 mL syringes (1 mL of 5.000 U/mL heparin). The 30 mL bone marrow-filled syringe was connected to a filter bag, to which 8 mL of Anticoagulant Citrate Dextrose (ACD-A) anticoagulant was added. Following homogenization, a new syringe was attached and the filtered 30 mL removed. The bone marrow aspirate was then transferred into specific process disposables, which were placed in a SmartPReP2 centrifuge. After 14 min of centrifugation, two phases were

		obtained within the tube, i.e., the plasma supernatant and the precipitated bone marrow cell concentrate. The plasma was removed using specific syringes provided in the kit; the cell concentrate was suspended and approximately 4 mL aspirated. The bone graft was mixed with bone marrow before placement at the site of the defect. The graft were then covered with an equine collagen membrane and the flaps were repositioned to completely cover the grafts and subsequently sutured with interrupted single 4–0 nylon sutures.
**Sinus Augmentation**		
**Nagata et al. 2012**	**Cell source:** Periosteum harvested from gingival connective tissue in the mandibular molar region<br>**Culturing/Expansion:** Yes<br>**Scaffold/Carrier:** Autogenous bone and PRP<br>**Time from harvesting to application:** Delayed – at least 6 weeks	Periosteum samples (50 mm2, 5×10 mm) were harvested from the molar region of the mandible under local anesthesia. Small pieces of the periosteum specimen were placed directly onto 100 mm culture dishes with culture medium (Medium 199 with Earle's salts, Invitrogen, Carlsbad, CA) containing 10% fetal bovine serum (sourced in New Zealand, SAFC Bioscience, Inc., Tokyo, Japan), 25 mg ascorbic acid (Sigma Chemical,St. Louis, MO), 100 IU/ml penicillin (Invitrogen), 100 µg/ml streptomycin (Invitrogen), and 250 ng/ml amphotericin B (Invitrogen) and incubated at 37 °C in an atmosphere of 10% CO2. Culture medium was changed every 3 days. Periosteum samples were incubated for around 6 weeks until the cells formed a sheet.<br>CAPC sheet fragments were mixed with PRP and particulate autogenous bone, and then with 2% CaCl2 (0.15 to 0.2 volume of PRP) to obtain a glue-like graft material in a few minutes . The amount of PRPs mixed was five to six times that of the bone weight of the grafts, but the composition of graft materials could not be strictly controlled due to differences in the amounts of harvested bone and CAPC sheets generated and also because of PRP glue formation.
**Prins et al. 2016**	**Cell source:** Stromal vascular fraction rich in adipose stromal/stem cells obtain from adipose tissue from the abdomen<br>**Culturing/Expansion:** No<br>**Scaffold/Carrier:** b-TCP particulate bone 0.7-1.4 mm, Bone ceramic (b-TCP (40%) + HA (60%) 0.5-1 mm<br>**Time from harvesting to application:** Immediate - hour (s)	Patients were brought under general anesthesia prior to the surgery. Adipose tissue was obtained manually from the abdominal wall using an aspiration 3-mm cannula with a Mercedes tip (Cloverleaf Medical) connected to a 60-ml Toomey syringe (GE Healthcare, Buckinghamshire, UK). Liposuction was continued until 150ml or more adipose tissue was harvested. The small surgical incisions were closed with intracutaneous absorbable Monocryl 5-0 (Ethicon; Johnson & Johnson International, Diegem, Belgium) and a pressure bandage (Tubigrip) was applied.<br><br>The syringes filled with adipose tissue were transported to a special stem cell laboratory within the VU University medical center operation complex. There, the adipose tissue was transferred to a Celution 800/CRS device (Cytori Therapeutics, Inc., San Diego, CA, USA) for automated and standardized extraction, washing, and concentration of the patient's own adipose-derived SVF according to the manufacturer's protocol (Celution 800/CRS software, version 4.1; Cytori Therapeutics, San Diego, CA), resulting in 5 ml of cell suspension. Viability and cell number were determined in triplicate with the release criterion set at >70% viability. Then $7.5x10^6$ cells per milliliter of Ringer's lactate solution were seeded onto 100% Ceros b-TCP with 60% porosity and granule size of 0.7–1.4 mm (Thommen Medical, Grenchen, Switzerland) or Straumann Bone Ceramic BCP, consisting of 60% hydroxyapatite and 40% b-TCP with 90% porosity and granule size of 0.5–1.0mm (Straumann, Basel, Switzerland).<br><br>For each maxillary sinus, 2 g calcium phosphate carrier with 20 x 10^6 cells (2.67 ml of the $7.5x10^6$ cells per milliliter) were prepared.<br>Cells were incubated on the carrier material for 30 minutes at room temperature. The cavity within the maxillary sinus was then filled with the calcium phosphate carrier with SVF, and the wound was closed with Vicryl Plus 3-0 sutures (Ethicon, Johnson & Johnson International).

**Ceccarelli et al. 2017**	**Cell source:** Periosteum derived stem cells obtained from gingival connective tissue samples<br>**Culturing/Expansion:** No<br>**Scaffold/Carrier:**<br>PLGA-Fisiograft / PLGA-HA los<br>**Time from harvesting to application:** Immediate - minutes	The grafts consisted of randomized biomaterial added to autologous micrografts obtained by mechanical disaggregation of a small portion of a gingival connective tissue sample (approx. 5 mm in length) which was collected directly from the surgical recipient site and washed with sterile saline; the sample was inserted in the Rigenera® filter to obtain the cellular graft(s) enriched with periosteum derived stem/progenitor cells.
**Kaigler et al. 2015**	**Cell source:** Bone marrow aspiration -Iliac crest<br>**Culturing/Expansion:** Yes<br>**Scaffold/Carrier:** b-TCP<br>**Time from harvesting to application:**<br>Delayed - At least 12 days	12 to 14 days before initial surgical treatment, 50 to 70 cc of bone marrow were aspirated from the posterior iliac crest. The collected marrow was transferred to a sterile blood bag and bone marrow mononuclear cells (BMMNC) were purified by Ficoll density gradient centrifugation. BMMNC were then inoculated into a bioreactor, which is a proprietary computer controlled, automated cell-processing unit, the Aastrom Replicell System (Aastrom Biosciences). This system incorporates single-pass perfusion in which fresh medium flows slowly over cells without retention of waste metabolites or differentiating cytokines. The culture medium consists of Iscove's modified Dulbecco's medium (IMDM), 10% fetal bovine serum, 10% horse serum, and 5 mM hydrocortisone. After cultivation for 12 days at 37 °C, 5% CO2, with a ramped continuous medium perfusion schedule, the ixymyelocel-t product was harvested by trypsinization, washed in a physiologic buffer, and collected into a sterile bag, where it was stored until the time of transplantation. the volume of cell suspensions delivered to each patient was determined by the volume of b-TCP used, and these volumes were mixed at a 1:1 ratio 30 minutes before delivery. The sinus cavity was then grafted under the elevated membrane by placing the b-TCP scaffold loaded with the cells. After placement of the graft, the sinus access window opening was then covered with a bioresorbable, occlusive collagen membrane, and the flap was sutured to attain primary closure.
**Sauerbier et al. 2011**	**Cell source:** Bone marrow aspiration - Pelvic bone<br>**Culturing/Expansion:** No<br>**Scaffold/Carrier:** BBM and Autogenous Thrombin<br>**Time from harvesting to application:** Immediate	The pelvic bone was punctured 2 cm laterocaudally from the superior posterior iliac spine. In a 60mL syringe, flushed with heparin solution (sodium-heparine, 10,000 U/mL, diluted with NaCl to 1000 U/mL; both from B. Braun) and then filled with 8mL of citric acid (BMAC-Kit; Harvest Technologies Corporation), 52mL of bone marrow was collected. Bone marrow cells were isolated in 15 min directly in the operating room by using the BMAC system (Bone Marrow Procedure Pack; Harvest Technologies Corporation). Sites were augmented with a combination of BBM (Bio-Oss® 0.25–1mm; Geistlich Pharma AG) and BMAC with autologous thrombin made from venous blood (Thrombin Kit; Harvest Technologies Corporation). The thrombin was needed to clot the BMAC solution around the BBM. Three milliliters of bone marrow concentrate and 1mL of autologous thrombin solution were added with a two-chamber syringe to 2 g of biomaterial with a volume of 4 cm3.20 The biomaterial was applied according to clinical needs. A collagen membrane (Bio-Gide®; Geistlich Pharma AG) was placed over the facial sinus wall to cover the graft. The mucoperiostal flap was replaced and closed with resorbable suture material (Vicryl 4-0; Ethicon).

**Periodontal regeneration**		
**Chen et al. 2016**<br>**Chen et al. 2018 (correction)**	**Cell source:** Periodontal ligament from extracted third molars<br>**Culture/Expansion:** Yes<br>**Scaffold/Carrier:** BBM (Bio-oss®)<br>**Time from harvesting to application:** Delayed: 4-5 weeks	The third molars were extracted and subjected to cell isolation and transplant production according to the Good Laboratory Practice and Good Manufacturing Practice (GMP) guidelines. The culture was prolongued until the cells grew to 80% confluence (7-10 days), at which time the cells were passaged. The number of cells at passage 4 was 1×10^7 (passage time: approximately 20 days).<br>To create the cell sheet, the PDLSCs will be digested by trypsin to obtain single cell suspensions and then inoculated on 6-well plates at 1×10^5 per well with L-ascorbic acid (vitamin C; VC, 30 µg/mL, Sigma) until confluent (approximately day 10). Next, he researchers observed changes in cell morphology and sheet-forming capacity. After a 10-day culture, white membranous substances appeared on the bottom of the wells. Before clinical application, the culture medium was discarded, and the cell sheets rinsed twice with phosphate-buffered saline (PBS; Gibco). Next, the 0.2-µm BBM particulates were distributed onto the surface of the cell sheets at a concentration of 0.25 g per well. The PDLSC cell sheets were then rolled up to pack the Bio-oss® particulates for clinical treatment. Bio-oss®/cell sheets (Cell group) were then administered to the bony defect region
**Ferraroti et al. 2018**	**Cell source:** Dental pulp from extracted tooth<br>**Culturing/Expansion:** No<br>**Scaffold/Carrier:** Collagen sponge (Condress®, Instituto Gentili, Italy)<br>**Time from harvesting to application:** Immediate: minutes	The tooth scheduled for extraction was removed, washed in 0.2% chlorhexidine (CHX) for 60 s prepared for DPSCs isolation.<br>The tooth was sectioned along the CEJ to expose the pulp chamber and the pulp tissue collected with a sterile Gracey curette. Then, the pulp was mechanically dissociated using the Rigenera Machine System (Rigenera®; HBW, Turin, Italy), a biological tissue disaggregator working at a rotating speed of 80 rpm, in 1.0 ml sterile physiologic solution. After dissociation, the cellular suspension was passed through a disposable grid (Rigeneracons) with 100 hexagonal holes filtering cells and component of extracellular matrix with a cut-off of 50 µm in an average time of 90 s. The obtained micrografts enriched in Dental pulp stem cells were endorsed onto a collagen sponge scaffold (Condress®, Istituto Gentili, Italy) to form a bio complex. The collagen sponge was placed to fill the defect, the flaps were repositioned and tension-free primary flap closure was obtained using horizontal internal mattress and interrupted sutures.
**Yamamiya et al. 2008**	**Cell source:** Periosteum from mandibular gingival connective tissue<br>**Culturing/Expansion:** Yes<br>**Scaffold/Carrier:** HA granules + PRP<br>**Time from harvesting to application:** Delayed: At least 6 weeks	Periosteum derived cell sheets were prepared by a trained specialist according to the Good Laboratory Practice and Good Manufacturing Practice (GMP) guidelines. After local anesthesia, periosteum samples approx.. 25 mm2 (5x5 mm) were harvested from the mandible. The periosteum specimens were placed directly onto 100-mm culture dishes with culture medium until the cells formed a sheet, which occurred after 6 weeks.<br>Fresh blood samples taken 1 week pre-operatively were used for obtaining PRP (stored at -80°C until used. Fifteen minutes prior to the use of the PRP in the periodontal surgical procedures, the PRP was rapidly thawed, and a coagulated preparation of 0.3 ml PRP was obtained by combining it with 0.1 g sodium alginate. Within a few minutes, the PRP preparation assumed a sticky gel consistency. Then, 0.5 g HA granules, with a particle size of 0.25 to 1.0 mm and a stomatal rate of 15%, were mixed with the coagulated PRP preparation. The coagulated PRP and HA mixture was placed, using amalgam condensers, into the defects to the vertical height of the corresponding adjacent bone level of the infrabony defect. Then, the cultured cell sheets were overlaid onto the PRP and HA granule mixture. The surgical flaps were repositioned to their presurgical levels and sutured with a 4-0 silk suture using an interrupted, vertical mattress technique.

Section/topic	#	Checklist item	Reported on page #
**TITLE**			
Title	1	Identify the report as a systematic review, meta-analysis, or both.	1
**ABSTRACT**			
Structured summary	2	Provide a structured summary including, as applicable: background; objectives; data sources; study eligibility criteria, participants, and interventions; study appraisal and synthesis methods; results; limitations; conclusions and implications of key findings; systematic review registration number.	2
**INTRODUCTION**			
Rationale	3	Describe the rationale for the review in the context of what is already known.	3
Objectives	4	Provide an explicit statement of questions being addressed with reference to participants, interventions, comparisons, outcomes, and study design (PICOS).	3-4
**METHODS**			
Protocol and registration	5	Indicate if a review protocol exists, if and where it can be accessed (e.g., Web address), and, if available, provide registration information including registration number.	3-4
Eligibility criteria	6	Specify study characteristics (e.g., PICOS, length of follow-up) and report characteristics (e.g., years considered, language, publication status) used as criteria for eligibility, giving rationale.	4 and Suppl
Information sources	7	Describe all information sources (e.g., databases with dates of coverage, contact with study authors to identify additional studies) in the search and date last searched.	4 and Suppl
Search	8	Present full electronic search strategy for at least one database, including any limits used, such that it could be repeated.	Suppl
Study selection	9	State the process for selecting studies (i.e., screening, eligibility, included in systematic review, and, if applicable, included in the meta-analysis).	Suppl
Data collection process	10	Describe method of data extraction from reports (e.g., piloted forms, independently, in duplicate) and any processes for obtaining and confirming data from investigators.	Suppl
Data items	11	List and define all variables for which data were sought (e.g., PICOS, funding sources) and any assumptions and simplifications made.	suppl
Risk of bias in individual studies	12	Describe methods used for assessing risk of bias of individual studies (including specification of whether this was done at the study or outcome level), and how this information is to be used in any data synthesis.	Suppl
Summary measures	13	State the principal summary measures (e.g., risk ratio, difference in means).	4 and Suppl

Synthesis of results	14	Describe the methods of handling data and combining results of studies, if done, including measures of consistency (e.g., I^2) for each meta-analysis.	Suppl

Section/topic	#	Checklist item	Reported on page #
Risk of bias across studies	15	Specify any assessment of risk of bias that may affect the cumulative evidence (e.g., publication bias, selective reporting within studies).	Suppl
Additional analyses	16	Describe methods of additional analyses (e.g., sensitivity or subgroup analyses, meta-regression), if done, indicating which were pre-specified.	Suppl
**RESULTS**			
Study selection	17	Give numbers of studies screened, assessed for eligibility, and included in the review, with reasons for exclusions at each stage, ideally with a flow diagram.	5
Study characteristics	18	For each study, present characteristics for which data were extracted (e.g., study size, PICOS, follow-up period) and provide the citations.	Suppl
Risk of bias within studies	19	Present data on risk of bias of each study and, if available, any outcome level assessment (see item 12).	7 and suppl
Results of individual studies	20	For all outcomes considered (benefits or harms), present, for each study: (a) simple summary data for each intervention group (b) effect estimates and confidence intervals, ideally with a forest plot.	Suppl
Synthesis of results	21	Present results of each meta-analysis done, including confidence intervals and measures of consistency.	8-11
Risk of bias across studies	22	Present results of any assessment of risk of bias across studies (see Item 15).	7 and suppl
Additional analysis	23	Give results of additional analyses, if done (e.g., sensitivity or subgroup analyses, meta-regression [see Item 16]).	8-11
**DISCUSSION**			
Summary of evidence	24	Summarize the main findings including the strength of evidence for each main outcome; consider their relevance to key groups (e.g., healthcare providers, users, and policy makers).	12-13
Limitations	25	Discuss limitations at study and outcome level (e.g., risk of bias), and at review-level (e.g., incomplete retrieval of identified research, reporting bias).	14-16
Conclusions	26	Provide a general interpretation of the results in the context of other evidence, and implications for future research.	16
**FUNDING**			
Funding	27	Describe sources of funding for the systematic review and other support (e.g., supply of data); role of funders for the systematic review.	1

From: Moher D, Liberati A, Tetzlaff J, Altman DG, The PRISMA Group (2009). Preferred Reporting Items for Systematic Reviews and Meta-Analyses: The PRISMA Statement. PLoS Med 6(7): e1000097. doi:10.1371/journal.pmed1000097

For more information, visit: **www.prisma-statement.org**.
